# Blood pressure variability in daily life: comparing individuals with cervical and upper-thoracic spinal cord injury to non-injured controls

**DOI:** 10.1038/s41371-026-01179-w

**Published:** 2026-07-09

**Authors:** Shane J. T. Balthazaar, Matthias Walter, Catherine R. Jutzeler, Andrea L. Maharaj, Andrei V. Krassioukov, Tom E. Nightingale

**Affiliations:** 1 School of Sport, Exercise and Rehabilitation Sciences, College of Life and Environmental Sciences, University of Birmingham, Edgbaston, Birmingham, UK; 2 Institute of Cardiovascular Sciences, University of Birmingham, Edgbaston, Birmingham, UK; 3 International Collaboration On Repair Discoveries (ICORD), Faculty of Medicine, University of British Columbia, Vancouver, BC Canada; 4 Department of Cardiology, University Hospitals Birmingham National Health Service (NHS) Foundation Trust, Birmingham, UK; 5 Faculty of Medicine, University of Basel, Basel, Switzerland; 6 Department of Health Sciences and Technology, ETH Zürich, Zürich, Switzerland; 7 SIB Swiss Institute of Bioinformatics, Lausanne, Switzerland; 8 Division of Physical Medicine and Rehabilitation, Faculty of Medicine, University of British Columbia, Vancouver, BC Canada; 9 GF Strong Rehabilitation Centre, Vancouver Coastal Health, Vancouver, BC Canada

**Keywords:** Cardiovascular diseases, Risk factors

## Abstract

**Background:**

Spinal cord injury (SCI) at or above the sixth thoracic spinal cord segment can disrupt autonomic cardiovascular control, leading to increased BP variability (BPV). This study compared BPV, nocturnal dipping patterns, and blood pressure (BP) fluctuations among individuals with cervical SCI (C-SCI), upper-thoracic SCI (UT-SCI), and non-injured controls (NI-C).

**Methods:**

Using 24-h ambulatory blood pressure monitoring, we analyzed BPV (via standard deviation [SD], coefficient of variation [CoV], average real variability [ARV], and variability independent of the mean [VIM]), nocturnal dipping, and systolic/diastolic BP fluctuations. Nocturnal dipping percentages were calculated and patterns classified. Hypotensive (SBP < 100 mmHg, DBP < 70 mmHg) and hypertensive (SBP > 150 mmHg per clinical guideline thresholds) events were identified. BP distribution was analyzed using skewness and kurtosis.

**Results:**

Eighty participants (44 C-SCI, 19 UT-SCI, 17 NI-C) had 66 ± 18 measurements taken and are included in the analysis. Overall 24-h BP levels were lower in the SCI groups than in NI-C. The C-SCI group exhibited significantly higher systolic BPV metrics across SD, CoV, ARV, and VIM compared to the UT-SCI and NI-C groups (*P* < 0.01). C-SCI nocturnal BP dips were reduced, and reverse dipping patterns were more prevalent (*P* < 0.001). A nadir-based trough nocturnal dipping analysis suggested reduced nighttime BP decline in C-SCI (*P* < 0.001). Hypotensive events occurred more frequently in C-SCI and UT-SCI compared to NI-C (both *P* < 0.001).

**Conclusion:**

Individuals with C-SCI showed significantly increased BPV and impaired nocturnal dipping, while both C-SCI and UT-SCI demonstrated heightened susceptibility to hypotensive events. These findings highlight the need for targeted cardiovascular monitoring and interventions in individuals with C-SCI and UT-SCI to mitigate the known risks associated with BP dysregulation.

## Introduction

Spinal cord injury (SCI) often leads to a complex array of impairments, encompassing both sensorimotor and autonomic dysfunction. [1–3] Disruption of autonomic cardiovascular control can lead to blood pressure (BP) abnormalities, [4] which may contribute to increased cardiovascular disease (CVD) risk in this population. [5] Injuries at or above the sixth thoracic spinal cord segment (T6) present challenges due to the loss or disruption of descending sympathetic control over the heart, trunk, and lower-extremity vasculature. [6] This disruption may lead to complications such as autonomic dysreflexia (AD) [7] and hypotensive episodes triggered by exercise, [8] orthostatic challenges, [9] or postprandial states. [10] The heart receives dual innervation – parasympathetic input via the vagus nerve and sympathetic input from T1-T5 segments of the spinal cord. [11] However, most blood vessels rely solely on sympathetic innervation, with the exception of blood vessels in the genitalia. [12] This selective innervation of target organs and variability in the degree of descending sympathetic vasomotor control contributes to unique cardiovascular responses and complexities in BP regulation within the SCI population. [13] Notably, some individuals with SCI remain asymptomatic during AD and hypotensive events, highlighting the importance of thorough assessment and management. [14] From a pathophysiological perspective, greater BP variability is expected in cervical SCI (C-SCI) due to loss of supraspinal sympathetic cardiac control compared with upper thoracic SCI (UT-SCI; T1-T6), where partial sympathetic cardiac regulation may persist.

Blood pressure naturally fluctuates in response to environmental, physical, and emotional stimuli. [15] While averaging multiple BP readings is a standard practice, this approach may not adequately capture the dynamic BP patterns observed in individuals with SCI, as autonomic dysregulation causes greater BPV, exaggerated responses to stimuli, and prolonged hypertensive or hypotensive episodes. [16–18] Blood pressure variability (BPV), defined as a change in the value of arterial blood pressure over a defined period of time, [19] has emerged as a critical parameter in cardiovascular research. Elevated BPV is associated with end-organ damage, stroke, and mortality, independent of mean BP. [20–22]

Ambulatory blood pressure monitoring (ABPM) provides valuable insights into BP lability in SCI, [23] revealing patterns with implications for cardiovascular morbidity and mortality. [24] Prior studies show disrupted circadian BP profiles, [25, 26] particularly among individuals with motor-complete tetraplegia. [24] In healthy individuals, BP typically declines by 10-20% during sleep due to reduced sympathetic activity. [27] Variations in nocturnal BP patterns include extreme dippers (BP drops >20%), dippers (BP drops 10-20%), non-dippers (BP drops <10%) and reverse dippers (BP increases at night). [28, 29] These classifications are clinically important, as reverse dipping is linked with increased CVD risk. [30]

Despite the potential of ambulatory BPV indices to capture differences in a free-living environment, their utility in a large SCI cohort compared with age-matched controls remains unclear. Frisbie and colleagues reported greater short-term BP instability in complete cervical SCI compared to low thoracic injuries, suggesting that higher injuries cause greater baseline BP volatility independent of posture or other triggers. [31] Similarly, Munakata and colleagues demonstrated attenuated or absent nocturnal BP dipping and exaggerated BP fluctuations in motor-complete tetraplegia, indicating profound autonomic dysregulation even without overt cardiovascular symptoms. [32] Rosado-Rivera and colleagues extended these findings by comparing 24-h BP and autonomic profiles across individuals with tetraplegia, high and low-level paraplegia, and controls, showing lower 24-h systolic BP and blunted nocturnal dipping in individuals with tetraplegia. [33] Collectively, these studies confirm that SCI alters circadian BP regulation and increases BP lability, but they have not comprehensively quantified multiple BPV indices in this population.

Therefore, this study expands on previous work by pooling data from four independent cohorts to enable stratification by neurological level of injury and comprehensive quantification of time-domain BPV metrics. While these indices (described in detail in the Methods) are well established in non-injured populations, [34] they remain underexplored in individuals with SCI. By integrating these indices, this study aims to clarify how injury level influences BPV patterns and to establish a foundation for assessing their potential prognostic relevance for cardiovascular risk in this population.

## Methods

### Study design and ethical considerations

This study is a secondary analysis of baseline data obtained from three longitudinal clinical trials (registration identifiers NCT02298660 [April 2013 – December 2017], [35] NCT02676154 [April 2016 – January 2019], [36] and NCT01718977 [April 2013 – April 2019], [37] and a cross-sectional study, all approved by the Clinical Research Ethics Board (CREB) at the University of British Columbia. Detailed information describing the objective of each of these trials and the trial specific inclusion and exclusion criteria can be found in Supplementary Table S1. Participants with SCI were assessed using the 2011 version of the International Standards for Neurological Classification of Spinal Cord Injury (ISNCSCI), a standardized neurological examination used to determine neurological level of injury (NLI) and classify the severity of motor and sensory impairment. [38] The severity of impairment was categorized according to the American Spinal Injury Association (ASIA) Impairment Scale (AIS), which ranges from AIS A (sensorimotor complete injury) to AIS D (sensorimotor incomplete injury). Participants were eligible if they were adults aged 18 to 60 years with a chronic ( > 1-year), traumatic SCI at or above T6. Both males and females were eligible to participate in the study, provided they were competent to give informed consent. Exclusion criteria for all participants in this pooled analysis included a history of cardiovascular or significant cardiopulmonary diseases, severe acute or unstable medical conditions, significant renal or hepatic dysfunction, recent major trauma or surgery within the past six months, or those with uncontrolled psychiatric or substance abuse issues. Additionally, women who were pregnant or breastfeeding were excluded. Age and sex-matched non-injured control (NI-C) data from the cross-sectional study were collected for comparison to the SCI participants.

### Ambulatory surveillance protocol

All enrolled participants underwent a 24-h ambulatory surveillance period, encompassing frequent episodic monitoring of brachial BP and HR (24-h ABPM) using either the Meditech Card (X)plore device (Meditech Ltd., Budapest, Hungary) or the Mobil-O-Graph (IEM, Stolberg, Germany), which have both been clinically validated for use. [23, 39] These devices have published protocol validations confirming the accuracy of brachial BP measurements (i.e., the basis for calculating time-domain BPV). [40] Although two device types were used across the pooled cohorts, all studies followed a uniform acquisition protocol and the same data analysis framework. Time-domain BPV indices are insensitive to fixed calibration differences, and standardizing sampling schedules and preprocessing steps helps minimize potential device-related effects. [41] BP and HR were set to record at least every 15 minutes from 9:00AM until 8:00PM, then at least hourly between 8:00PM to 9:00AM, with adjustments based on participants’ self-reported sleep and wake times. Across all study protocols, participants (C-SCI, UT-SCI, and NI-C) were also encouraged to take additional BP and HR readings at any time if they experienced any symptoms of low or high BP or engaged in any activities that might influence BP (e.g., urinary bladder and bowel management, physical exertion, moving to an upright position, taking medications, eating, etc). Inconsistencies in how thoroughly the logs were completed prevented a reliable analysis of whether events were clustered around identifiable activities. However, scheduled (i.e., automatic) versus participant-triggered (i.e., manual) readings were identified. To summarize each participant’s typical BP over 24 h, median 24-h systolic BP (SBP) and diastolic BP (DBP) were calculated from all valid ambulatory readings. As ambulatory BP in chronic SCI is often skewed and characterized by episodic excursions, the median provides a robust participant-level measure of central tendency that is less influenced by transient extremes. These extreme values were not removed from analysis; they were incorporated into all within-participant BPV indices, which were computed from the full time series. As a sensitivity check, all analyses were repeated using the mean 24-h SBP and DBP, with no material change in results. Considering the real-world challenges of ambulatory BP monitoring in the SCI population, [24, 42] a valid measurement was defined as having at least 60% of attempted BP readings successfully recorded (i.e., an attempted BP measurement that gives an accurate reading and not an error warning) during the 24-h monitoring period. This threshold ensured sufficient data to reliably represent each participant’s BP patterns and allow for meaningful analysis. To assess BPV, which has been used as a marker for CVD risk in prior studies, statistical measures such as the standard deviation (SD) of an individual’s BP readings can be used. [43, 44] Other measures of variability include the coefficient of variation (CoV), average real variability (ARV), and the variability independent of the mean (VIM). [15] CoV, the ratio of SD to the mean, is more sensitive to lower BP values and is used as a method to mitigate the influence of mean arterial BP on mortality. [45] ARV, derived from the differences between consecutive BP readings and averaged by dividing the sum by the number of measurements minus one, has shown promise as a superior measure of BPV. [46, 47] While shorter durations of ARV provide valuable insights with continuous beat-by-beat monitoring, [48] full 24-h monitoring provides a more comprehensive assessment of daily BPV outside of the laboratory. VIM measures variability uncorrelated with mean BP by adjusting the SD of BP with the power of the mean. [49] We calculated these four BPV indices to provide a comprehensive profile of variability, as each measure captures different statistical and physiological aspects of BP fluctuations. While ARV is widely used in ABPM research for its ability to quantify changes between consecutive readings, [50] it does not fully capture all variability patterns relevant to SCI. Therefore, we did not designate a single “primary” BPV outcome, but for the purposes of this manuscript, considered these four indices equally important and complementary. This multi-index approach provides a more comprehensive clinical interpretation and facilitates both comparison across participant groups and alignment with previous work that used different BPV metrics.

### Nocturnal Dip

As individuals with chronic SCI often experience sleep-onset BP perturbations due to transfers, care routines, and AD-related episodes, [51] we evaluated nocturnal dip for SBP and DBP using a nadir-based trough approach by averaging the single lowest nighttime SBP or DBP value with its adjacent nighttime readings. This three-reading nighttime trough average was then subtracted from the median SBP and DBP values obtained during the day. The percentage was determined by dividing the difference between these average nadir-based nighttime readings and the median daytime BP by the median daytime BP, then multiplying by 100. This approach was used to characterize the lowest nighttime BP period rather than the average sleep-period BP. Inferential conclusions from this non-standard approach are interpreted cautiously. As a sensitivity analysis, we also calculated conventional nocturnal dipping using participant-specific daytime and nighttime periods as coded in the dataset. [52] In this analysis, daytime and nighttime SBP and DBP were defined as the mean BP during the awake and sleep periods, respectively, and dip percentage was calculated as: Dip (%) = ([daytime BP − nighttime BP] / daytime BP) × 100. [52]

Participants were sub-classified according to the percentage of nighttime BP decline as follows: extreme dippers if nighttime BP reduction ≥20%; dippers if the decline was between 10-20%; non-dippers if the decline was between 0-10%; and the reverse dippers if there was an increase in nighttime BP. [53] As this approach is non-standard, inferential conclusions from dipper categories are interpreted with caution and are exploratory.

### Blood pressure fluctuations

Individuals with SCI at or above T6 frequently experience AD and hypotensive episodes, [8, 10, 54, 55] leading to pronounced BP fluctuations that contribute to increased BPV. We classified hypotensive events as SBP < 100 mmHg and DBP < 70 mmHg, [56] excluding nighttime BP (to avoid misclassifying nocturnal dip as hypotensive events). Because individuals with chronic SCI commonly have resting BP values below traditional clinical cut-offs, these absolute thresholds were applied to quantify hypotensive events in a manner appropriate for the SCI population, rather than to diagnose hypotension based on general-population criteria. Hypertensive events were defined as SBP > 150 mmHg, reflecting a substantial deviation from typical resting SBP values in individuals with C-SCI, which often range from 90-110 mmHg. [57] This threshold aligns with the value recommended by the Consortium of Spinal Cord Medicine for pharmacological management of a refractory episode of AD. [58] Although no universally accepted BP threshold defines immediate danger in SCI, the use of absolute cutoffs is consistent with existing clinical guidelines and commonly used criteria. This allows for consistent classification of BP events across participants and captures the extreme fluctuations that contribute to increased BPV. [59]

### Statistical analysis

Group data are expressed as mean ± standard deviation. Demographics, participant-level median 24-h SBP and DBP, along with participant-level BPV metrics for SBP and DBP (i.e., SD, CoV, ARV, and VIM) were summarized and statistically compared between groups using analysis of variance (ANOVA) with Bonferroni post hoc (to account for different types of variables) or Kruskal Wallis with Mann-Whitney U post hoc, if non-parametric. In addition to Bonferroni-adjusted post hoc tests, false discovery rate (FDR) control using the Benjamini–Hochberg procedure (α = 0.05) was applied separately across the four systolic and four diastolic BPV indices to account for multiple testing across indices. Eta squared (η^2^) was calculated to determine effect size and was defined as small (η^2^ ≥ 0.01), medium (η^2^ ≥ 0.06), and large (η^2^ ≥ 0.14) effects. [60] Tests were two-sided with a significance level of 0.05, unless otherwise specified. Statistical analyses were performed using standard statistical packages in R (Version 4.4.1, R Foundation for Statistical Computing, Vienna, Austria). Graphical representations were created in R Studio (Version 2024.09.0, Integrated Development Environment for R. Posit Software, PBC, Boston, MA, USA) and Adobe Illustrator (Version 28.1, Adobe Inc., 2023, San Jose, CA, USA).

BPV was assessed using SD (mean difference from mean BP), CoV (SD divided by mean), ARV (mean of absolute successive differences), and VIM (adjustment of SD using a power transformation of the mean BP). The prevalence of SBP and DBP dipping patterns (i.e., extreme dippers, dippers, non-dippers, and reverse dippers) [28, 29] were compared across the C-SCI, UT-SCI, and NI-C groups using chi-square (*χ*^2^) tests.

The relationship between BP lability and NLI was examined using participant-level negative binomial regression models. The prevalence of hypotensive and hypertensive events was assessed by using chi-square tests on the participants in each group. We further examined the distributional characteristics of the ABPM data by calculating the skewness and kurtosis of each participant’s ABPM recordings to further describe BP differences between groups. Skewness indicates the symmetry of a distribution, with zero showing perfect symmetry, while kurtosis measures the relative heaviness of the tails of a distribution, with higher values indicating heavier tails and greater departure from normality, rather than necessarily more outliers. Negatively skewed distributions have tails toward lower values, and leptokurtic distributions have thicker tails and increased BP lability. [56] The z-scores for skewness and kurtosis were also calculated to characterize the magnitude of the deviations from normality for each participant by group. [61]

An unbiased recursive partitioning conditioning inference tree (URP-CTREE), a regression model that conducts sequential tests of independence among predictors (biological sex, age, Time Since Injury (TSI), weight, NLI, AIS, and each research study), was utilized to explore the link between these predictors and 24-h ABPM indices. A more detailed description of URP-CTREE is provided in the cited methodological reference. [62]

To assess the robustness of our pooled analysis, we performed sensitivity analyses by examining the SCI cohorts between the four studies. Specifically, we conducted one-way ANOVAs to evaluate the impact of the sub-studies on SBP of SD, CoV, ARV, and VIM. Post hoc pairwise comparisons were conducted using Tukey’s HSD test (for Type II error control and more power) to explore significant differences between subgroups.

As BP was recorded every 15 minutes during the daytime and at least hourly overnight, we evaluated whether unequal sampling density biased BPV estimates. To test this, we performed a sensitivity analysis in which daytime BP data were “hourly-thinned” by aggregating all readings within each hour to their median SBP value. Participants were included if their number of retained daytime hourly bins was at least equal to their number of valid nighttime hourly bins. BPV indices (SD, CoV, ARV, and VIM for SBP) were recalculated using this reduced dataset. This conservative approach further ensured that variability estimates were not inflated by clusters of irregularly spaced variability measurements (e.g., from symptom-triggered readings). We note that previous work has discussed the importance of consistent measurement intervals and sampling frequency in BPV research, [63, 64] however we attempted to account for the known acute, episodic BP surges and drops common in SCI, which can produce clusters of readings and artificially elevate time-domain BPV metrics. To our knowledge no standard protocol describes “hourly-thinning” as we have done to account for this unequal sampling. Therefore, this procedure is presented as a supplemental sensitivity analysis, and the results are reported alongside the primary dataset. In an additional sensitivity analysis, systolic BPV indices were recalculated using scheduled readings only to assess whether the main findings were disproportionately influenced by participant-triggered measurements.

## Results

Data were collected from 80 participants (STARD Flow Diagram; Supplementary Figure S1), including 44 with C-SCI, 19 with UT-SCI, and 17 NI-C. The cohort was predominantly male (80%). The measurement ratio, defined as the number of successful brachial BP recordings out of the pre-programmed attempts, showed no significant differences among the groups. Likewise, the total number of measurements taken over 24 h did not differ significantly. Approximately 80% of measurements were protocol-scheduled (automatic) and 20% were participant-triggered (manual), with no significant difference in participant-triggered readings between groups (Kruskal-Wallis χ² = 0.63, *P* = 0.73). The groups were also comparable in terms of age, weight, and time since injury (TSI; for participants with SCI). All data are summarized in Table 1.

**Table 1:** Participant Characteristics.

Outcome	C-SCI (*n* = 44)	UT-SCI (*n* = 19)	NI-C (*n* = 17)	*P* value
Age [years], mean ± SD	43 ± 10	43 ± 13	41 ± 15	0.77
Sex (M), n (%)	34 (77%)	16 (84%)	14 (82%)	0.79
Body mass [Kg]	75 ± 20	77 ± 14	82 ± 13	0.42
NLI	C1 = 1		-	-
	C4 = 8	T2 = 3		
	C5 = 19	T3 = 5		
	C6 = 11	T4 = 6		
	C7 = 4	T5 = 4		
	C8 = 1	T6 = 1		
AIS Grade	A = 20		-	-
	B = 18	A = 18		
	C = 4	B = 1		
	D = 2			
TSI [years]	15 ± 9	17 ± 14	-	0.51
Measurement Ratio	87 ± 12	86 ± 18	86 ± 14	0.95
Total Number of Successful Measurements (automatic + manual)	67 ± 17	66 ± 23	65 ± 15	0.93
Participant-triggered measurements, % of successful readings	20 ± 11	19 ± 11	25 ± 22	0.73

*AIS* american spinal injury association (ASIA) impairment scale, *C-SCI* cervical spinal cord injury, *M* male, *NI-C* non-injured control, *NLI* neurological level of injury, *TSI* time since injury, *UT-SCI* upper-thoracic spinal cord injury.

AIS categories: A, sensorimotor complete injury; B, sensory incomplete, motor complete; C, sensory incomplete, motor incomplete with >50% of key muscles below the neurological level having no active movement against gravity; D, sensory incomplete, motor incomplete with ≥50% of key muscles below the neurological level having at least active movement against gravity. Injury levels: C, cervical spinal cord segments; T, thoracic spinal cord segments.

### Blood pressure variability indices

**Fig. 1:**
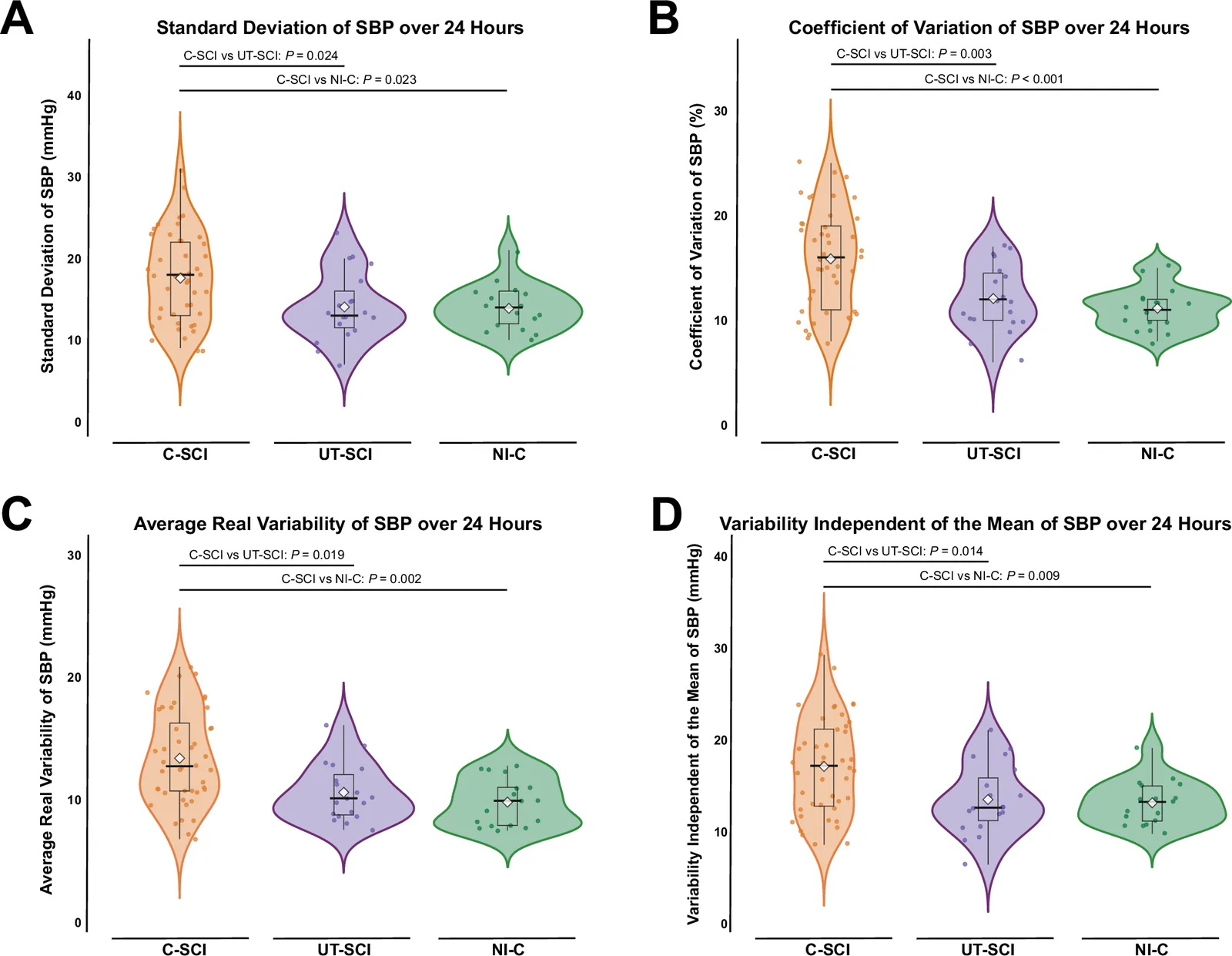
Comparison of systolic blood pressure variability indices over a 24-h period. **A** Standard deviation (SD) for systolic blood pressure (SBP) over 24-h, **(B)** Coefficient of Variation (CoV) for SBP over 24-h, **(C)** Average Real Variability (ARV) for SBP over 24-h, **(D)** Variability Independent of the Mean (VIM) for SBP over 24-h. Violin plots are presented with individual participant data points with box-and-whisker plots overlaid; the thick horizontal black line represents the median value of the respective variable and the white diamond represents the mean. Orange represents the cervical spinal cord injury (C-SCI) group, purple represents the upper-thoracic spinal cord injury (UT-SCI) group, and green represents the non-injured control group (NI-C). Statistical significance using a Bonferroni-adjusted post hoc test is shown if the one-way ANOVA (normal distribution) or Kruskal-Wallis (non- normal distribution) for comparison across all groups was significant. See Supplementary Data for detailed statistics.

Standard deviation for SBP was significantly (*P* = 0.004, η^2^ = 0.130) different across groups, being significantly higher in the C-SCI group (18 ± 5 mmHg) compared to the UT-SCI (14 ± 4 mmHg) and NI-C (14 ± 3 mmHg) groups. CoV for SBP was significantly (*P* < 0.001, η^2^ = 0.227) different across groups, being significantly higher in the C-SCI group (16 ± 5%) compared to the UT-SCI (12 ± 3%) and NI-C (11 ± 2%) groups. ARV for SBP was significantly (*P* < 0.001, η^2^ = 0.209) different across groups, being significantly higher in the C-SCI group (13 ± 4 mmHg) compared to the UT-SCI (11 ± 2 mmHg) and NI-C (10 ± 2 mmHg) group. VIM for SBP was significantly (*P* = 0.002, η^2^ = 0.154) different across groups, being significantly higher in the C-SCI group (17 ± 5 mmHg) compared to the UT-SCI (14 ± 4 mmHg) and NI-C (13 ± 2 mmHg) group (Fig. 1).

Effect sizes indicated large effects of neurological level of injury on systolic BPV indices (η^2^ = 0.27-0.34). After FDR correction across indices, all four systolic indices (SD, CoV, ARV, and VIM) remained significant (q ≤ 0.002). For diastolic BPV, only CoV remained significant after FDR correction (q = 0.0496; η^2^ = 0.12).

### Median SBP and DBP and nocturnal Dip

**Fig. 2:**
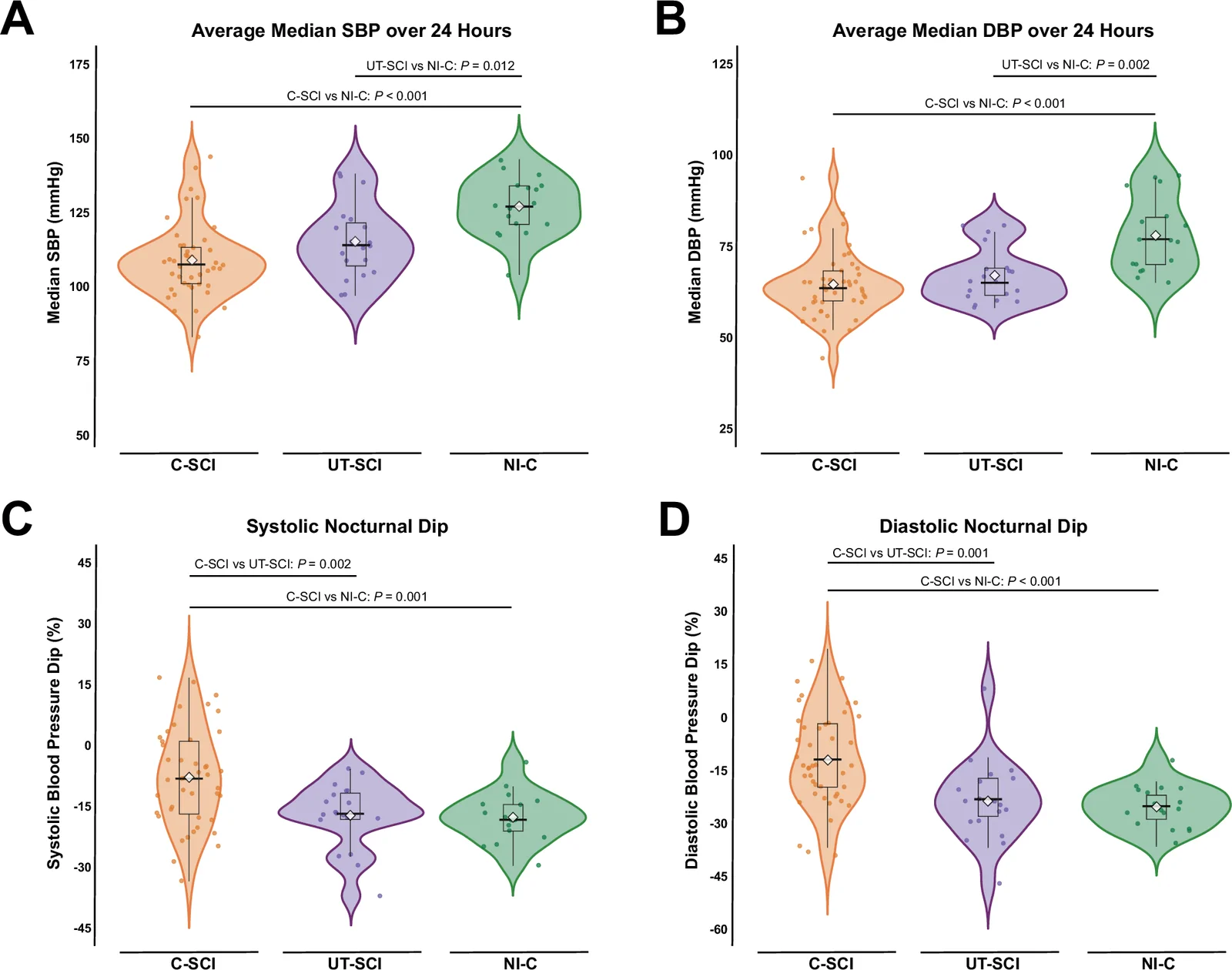
Comparison of median blood pressure readings over a 24-h period with assessment of nocturnal dip. **A** Mean of the median systolic blood pressure (SBP) over 24-h, **(B)** Median diastolic blood pressure (DBP) over 24-h, **(C)** Systolic blood pressure nocturnal dip over 24-h, **(D)** Diastolic blood pressure nocturnal dip over 24-h. Violin plots are presented with individual participant data points with box-and-whisker plots overlaid; the thick horizonal black line represents the median value of the respective variable and the white diamond represents the mean. Orange represents the cervical spinal cord injury (C-SCI) group, purple represents the upper-thoracic spinal cord injury (UT-SCI) group, and green represents the non-injured control group (NI-C). Statistical significance using a Bonferroni-adjusted post hoc test is shown if the one-way ANOVA (normal distribution) or Kruskal-Wallis (non- normal distribution) for comparison across all groups was significant. See Supplementary Data for detailed statistics.

The mean of the median SBP over 24 h was significantly (*P* < 0.001, η^2^ = 0.268) different across groups, being significantly higher in the NI-C group (127 ± 10 mmHg) compared to the C-SCI (109 ± 13 mmHg) and UT-SCI (115 ± 12 mmHg) group. The mean of the median DBP over 24 h was significantly (*P* < 0.001, η^2^ = 0.259) different across groups, being significantly higher in the NI-C group (78 ± 10 mmHg) compared to the C-SCI (65 ± 9 mmHg) and UT-SCI (67 ± 7 mmHg) group. Nocturnal SBP dip was significantly (*P* < 0.001, η^2^ = 0.221) different across groups, being significantly reduced in the C-SCI group (-6 ± 12%) compared to the UT-SCI (-17 ± 8%) and NI-C (-18 ± 7%) group. Nocturnal DBP dip was significantly (*P* < 0.001, η^2^ = 0.262) different across groups, being significantly reduced in the C-SCI group (-10 ± 14%) compared to the UT-SCI (-23 ± 11%) and NI-C (-26 ± 6%) group (Fig. 2). In a conventional daytime versus nighttime sensitivity analysis, conventional SBP dip remained significantly different across groups, with dips of -2 ± 10% in C-SCI, -13 ± 9% in UT-SCI, and 15 ± 5% in NI-C. Conventional DBP dip showed the same pattern, with mean dips of -6 ± 11%, -18 ± 12%, and -21 ± 6%, respectively. Therefore, the overall interpretation of impaired nocturnal BP decline in C-SCI was unchanged. A greater proportion of reverse dippers was observed in the C-SCI group compared to the UT-SCI or NI-C groups for both SBP and DBP (Supplementary Figure S2).

### Blood pressure lability

**Fig. 3:**
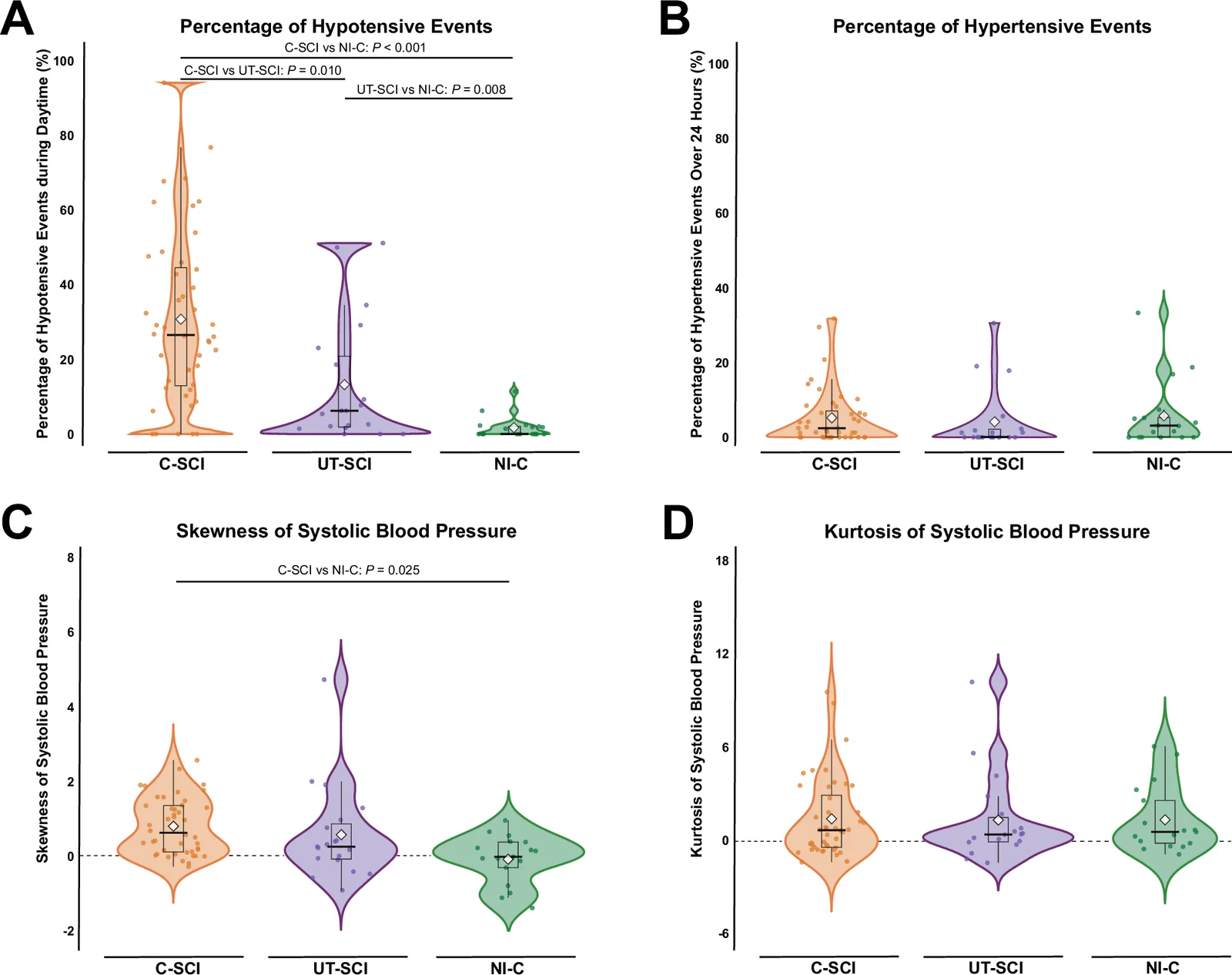
Comparing hypotensive and hypertensive events across different injury characteristics. **A** The percentage of hypotensive events for each participant over the 24-h ABPM recording period categorized by neurological level of injury (NLI), **(B)** The percentage of hypertensive events for each participant over the 24-h ABPM recording period categorized by NLI, **(C)** The skewness of the SBP values were calculated for each participant independently, **(D)** The kurtosis of the SBP values were calculated for each participant independently. Violin plots are presented with individual participant data points with box-and-whisker plots overlaid; the thick horizonal black line represents the median value of the respective variable and the white diamond represents the mean. Orange represents the cervical spinal cord injury (C-SCI) group, purple represents the upper-thoracic spinal cord injury (UT-SCI) group, and green represents the non-injured control group (NI-C). Statistical significance using a Bonferroni-adjusted post hoc test is shown if the one-way ANOVA (normal distribution) or Kruskal-Wallis (not normal distribution) for comparison across all groups was significant. See Supplementary Data for detailed statistics.

The percentage of brachial BP recordings that met the criteria for hypotensive or hypertensive events are shown in Fig. 3A, B, respectively, to account for the different number of measurements across participants. Participant-level negative binomial models were used to examine event counts, with the total number of eligible recordings included as an exposure term and NI-C as the reference category. For hypotensive events, compared with NI-C, the incidence-rate ratio was 19.57 (95% CI 9.23 to 41.50; P < 0.001) for C-SCI and 8.71 (95% CI 3.77 to 20.13; P < 0.001) for UT-SCI. For hypertensive events, the corresponding incidence-rate ratios were 0.91 (95% CI 0.37 to 2.20; *P* = 0.832) for C-SCI and 0.72 (95% CI 0.25 to 2.05; *P* = 0.541) for UT-SCI. The number of hypotensive and hypertensive events can be found in Supplementary Table S2.

For hypotension, the effect of group was significant, with the highest burden observed in the C-SCI group. The mean percentage of daytime hypotensive readings was 31% in C-SCI, 13% in UT-SCI, and 2% in NI-C, indicating that individuals with higher-level SCI exhibit a substantial burden of low BP during the daytime period.

The SBP skewness distribution (Fig. 3C) differed significantly across groups (*P* = 0.004, η² = 0.136), being markedly right-skewed in C-SCI (0.80 ± 0.76) and moderately skewed in UT-SCI (0.58 ± 1.27) compared with a near-symmetrical NI-C distribution (-0.08 ± 0.65). SBP kurtosis (Fig. 3D) did not differ significantly between groups (*P* = 0.10, η² = 0.023). Z-score analysis further revealed that 50% of the C-SCI group and 42% of the UT-SCI group exhibited significantly non-Gaussian SBP distributions, compared with 35% of NI-C.

The URP-CTREE analysis was used to identify subgroups by NLI (C-SCI, UT-SCI, NI-C), TSI, age, sex, and study identifier to assess systolic BPV indices (SD, CoV, ARV, VIM) over 24 h. NLI emerged as the single, strongest predictor, accounting for a moderate proportion of the variance in BPV (R^2^: 0.26-0.32). Conditional inference trees illustrating the BPV trends are provided in Supplementary Figure S3.

### Sensitivity analysis

The one-way ANOVA for SBP did not reveal significant differences between the SCI trials for SD (*P* = 0.49), CoV (*P* = 0.57), ARV (*P* = 0.19), and VIM (*P* = 0.17). No significant differences for these outcomes between studies supports the validity of pooling these datasets despite minor differences in the eligibility criteria of the original study protocols. Sensitivity analyses using the hourly-thinned daytime dataset (median within hour) produced qualitatively similar results to the full 24-h dataset (Supplementary Table S3). This indicates that the elevated BPV observed in C-SCI is not an artifact of unequal daytime/nighttime sampling frequency.

## Discussion

Our results demonstrate significant alterations in ambulatory BP components among individuals with SCI, particularly those with C-SCI, highlighting notable cardiovascular dysregulation in this population (*the central finding of this study*). Elevated BPV, a known predictor of cardiovascular morbidity and mortality in non-SCI populations, [65] warrants investigation as a potential prognostic marker in individuals with SCI. Although our findings reveal clear alterations in BP components, longitudinal studies are required to determine whether these BPV abnormalities translate into increased cardiovascular risk in this population.

### Blood pressure variability

Time-domain BPV indices, including SD, CoV, ARV, and VIM, were significantly elevated in the C-SCI group compared to UT-SCI and NI-C. Complementary analyses, such as unbiased recursive conditional inference trees (Supplementary Figure S3), identified NLI as the primary determinant of BPV, independent of other factors such as TSI or AIS severity. Elevated BPV in individuals with C-SCI likely reflects impaired autonomic cardiovascular regulation, [66] emphasizing the need for targeted BP management strategies in this specific group. Unlike the current focus of achieving normotensive BP targets, [67, 68] our findings suggest that reducing BPV, particularly in those with high-level chronic SCI, may also be important as previous work in the general population has linked higher BPV to increased risk of cardiovascular events. [69]

A study of over 42,000 non-SCI adults reported that BPV reliably predicts cardiovascular outcomes and is associated with increased risk for new onset of CVD, such as myocardial infarction, stroke, and heart failure. [70] This association persisted across various age groups, sexes, and clinical comorbidities. Our data further indicates that individuals with C-SCI exhibit a much wider range of BPV compared to NI-C, characterized by more pronounced BP fluctuations and the absence of a normal nocturnal dip in BP.

Despite the increasing interest in BPV metrics, few studies have explored these measures in the SCI population. Consistent with our findings, another study found that individuals with C-SCI (n = 33) and UT-SCI (n = 3) experience significant BP instability, showing frequent asymptomatic fluctuations between hypertension and hypotension episodes compared to ambulatory NI-C (n = 13). [14] In terms of CoV for SBP during the day (i.e., awake period), our data (Supplementary Table S2) reported 16 ± 5% for C-SCI, 11 ± 3% for UT-SCI, and 10 ± 2% in NI-C (*P* < 0.001), similar to the values of 15 ± 1% for SCI versus 10 ± 1% for NI-C (*P* = 0.0013) reported by Wang and colleagues. [14] ARV for SBP during the day also mirrored these findings, with our study showing 14 ± 4 mmHg for C-SCI, 11 ± 3 mmHg for UT-SCI, and 10 ± 2 mmHg for NI-C (*P* < 0.001), compared to 13 ± 1 mmHg for SCI and 10 ± 1 mmHg for NI-C. Our study’s use of data from multiple studies, resulting in a larger number of UT-SCI participants, allowed for a more thorough stratification by NLI, providing more insight into its role in cardiovascular dysregulation, which was not addressed in the previous study. [14] Additionally, while the other study focused on fewer BPV indices, our study examined both SD and VIM, offering for the first time a more comprehensive evaluation of BPV in individuals with SCI.

The extent to which mechanisms underlying elevated BPV in individuals with SCI mirror those observed in non-injured individuals with cardiovascular disease remains poorly understood. In non-SCI populations, elevated BPV has been associated with arterial stiffness, impaired baroreflex sensitivity, endothelial dysfunction, and systemic inflammation, each of which has been implicated in long-term cardiovascular risk. Importantly, many of these abnormalities have also been independently documented in individuals with SCI. [71–74] In contrast to essential hypertension, the ambulatory phenotype observed after high-level SCI is characterized by lower overall BP together with marked instability, impaired circadian regulation, and the burden of a high hypotensive load during daytime. Injury-related autonomic dysregulation is likely a major contributor to this pattern, although vascular and endothelial abnormalities have also been documented after SCI. [72, 75] Longitudinal studies are needed before BPV metrics can be interpreted as risk markers in individuals the same way they are used in non-injured populations.

Systolic BPV, which is linked to arterial stiffness and aging in non-SCI populations, [76] may be particularly relevant for individuals with SCI, given that this condition is often described as a model of premature aging. [71, 73, 77] Evidence indicates that individuals with SCI, especially those with paraplegia, exhibit higher aortic pulse wave velocity (aPWV; a marker of arterial stiffness), compared to individuals with tetraplegia, [78] although lower resting BP in tetraplegia may partially influence these findings. [76] Diastolic BPV (see Supplementary Table S2) has been associated with autonomic and endothelial dysfunction. [76] Endothelial dysfunction is also exaggerated in the SCI population. [72] Our prior work further supports the presence of persistent central arterial stiffness in SCI, even when individuals are well matched with NI-C. [73] Together, these observations suggest that BPV in SCI arises from the interaction between autonomic impairment and vascular remodelling. Longitudinal studies are required to clarify the role of BPV in risk stratification, whether therapeutic interventions (such as exercise [79]) can modulate BPV and to inform targeted interventions aimed at reducing cardiovascular risk in individuals with SCI more generally.

### Blunted nocturnal dipping

Individuals with C-SCI demonstrated a markedly attenuated nocturnal dip compared to UT-SCI and NI-C. Consistent with prior observations in SCI, [80, 81] many participants with C-SCI exhibited non-dipping or reverse-dipping patterns, reflecting substantial disruption of autonomic cardiovascular regulation in this group. In contrast, UT-SCI participants showed more preserved, though still variable, nocturnal BP declines.

Although abnormal nocturnal BP patterns (for non-dipping and reverse dipping) have been associated in the general population with adverse cardiovascular markers such as stroke risk, subclinical organ damage, and mortality, [82–87] our study did not assess cardiovascular outcomes or intermediate CVD biomarkers. Furthermore, nocturnal dipping patterns should be reported in the context of overall BP level. In individuals with chronic SCI who are overtly hypotensive, nocturnal dip does not necessarily reflect physiological cardiovascular recovery during sleep, as it might in normotensive or hypertensive populations. For example, a 20% nighttime BP reduction in a participant with a mean SBP of 90 mmHg would represent profound hypotension rather than a healthy circadian rhythm. Thus, conventional dipper/non-dipper classifications may not accurately capture cardiovascular health in individuals with chronically low baseline BP, and alternative or adjusted interpretative frameworks may be warranted in this population.

Nevertheless, given the established associations between circadian BP disruption and cardiovascular vulnerability in other populations, [83, 86–89] the high prevalence of blunted or reversed nocturnal dipping in C-SCI emphasizes the degree of autonomic impairment in this group. Whether restoring more physiological nocturnal BP patterns could improve cardiovascular health in SCI remains unknown and warrants further investigation. These findings should be viewed as descriptive evidence of altered BP regulation rather than as clinical prognostic indicators.

### Altered blood pressure dynamics in SCI

Participants with C-SCI and UT-SCI exhibited profound BP regulation impairments, characterized by a higher frequency of hypotensive events compared to NI-C. The proportion of hypotensive readings was over six-fold higher in C-SCI than in controls (31% vs 5%). This pattern likely reflects impaired sympathetic vasoconstrictor control and blunted baroreflex buffering following disruption of descending sympathetic pathways. [90] This high hypotensive load may exacerbate the degree of orthostatic, postprandial, and post-exercise hypotension in individuals with SCI, thereby resulting in chronic end-organ hypoperfusion. [91] Given the lower resting BP commonly observed in SCI, even modest additional BP reductions may be sufficient to cross cerebral perfusion thresholds and provoke hypotensive symptoms, [92] whereas comparable relative BP reductions in individuals with higher baseline BP may remain asymptomatic.

Although hypertensive events were not directly associated with NLI in our study, they remain important to monitor, particularly to capture transient spikes such as those caused by AD. [16] Elevated SBP increases cardiovascular workload and accelerates arterial damage, leading to an increased myocardial infarction and stroke risk. [93] Since SBP is a more reliable predictor of future cardiovascular risk than DBP, [94, 95] targeting systolic BPV indices may be more important in mitigating cardiovascular risks in the SCI population.

In addition to event frequency, our analysis of distributional characteristics revealed that BP patterns in SCI are often non-Gaussian (not normal) in shape, particularly in the C-SCI group. Using a z-score-based detection of skewness and kurtosis, we found 50% of C-SCI and 42% of UT-SCI participants in our cohort demonstrated significantly skewed or heavy-tailed SBP distributions, indicating a higher prevalence of extreme SBP values and BP lability. Such distributional abnormalities may represent greater end-organ stress, as repeated transient spikes or drops in BP can damage the vascular endothelium, even when mean BP appears within normal limits. [96] These findings align with previous work showing increased skewed SBP distributions in individuals with tetraplegia and non-Gaussian profiles in upper-thoracic injuries. [56] However, ambulatory BP distributions may be asymmetric rather than Gaussian and are shaped by circadian variation as well as activity- and sleep-related changes. [97, 98] In this context, the greater skewness observed in SCI should be interpreted cautiously as descriptive evidence of altered BP patterns. Therefore, z-score-based detection may also provide complementary descriptive information on BP instability that may not be captured by daily mean or median BP values alone.

### Clinical considerations and future directions

From a practical standpoint, these findings support the routine use of 24-h ABPM in individuals with high-level SCI to capture BP fluctuations that are frequently not captured during a typical clinic visit with the current standard of averaging multiple BPs. Clinicians should interpret BP patterns beyond mean or median values, paying attention to variability indices and nocturnal dipping profiles. Identifying individuals with exaggerated BPV or reverse dipping may help guide individualized management, though standardized thresholds for BPV in SCI are not established. BPV impacts vascular endothelial cells via altered wall shear stress, exacerbating atherosclerotic plaque formations. [99] This accelerated plaque formation occurs though molecular pathways, including ion channel activation and DNA changes. Proteins like Piezo1, integral to cardiovascular regulation, are also affected, promoting plaque development via the NF-кB pathway. [100] These insights emphasize the importance of stabilizing BPV as a therapeutic target to reduce atherosclerosis progression.

While our study highlights the role of BPV in cardiovascular risk, the inclusion of concurrent arterial stiffness assessments or inflammatory markers would have strengthened our findings, allowing for a more direct link between BPV and established prognostic risk factors, facilitating a more comprehensive CVD risk stratification. However, addressing BPV through comprehensive assessments, including 24-h ABPM, may improve cardiovascular risk stratification and management for these individuals.

Although unnoticed by many individuals with SCI, [14] SBP variability and arterial stiffness are more strongly associated with the risk of stroke [101] and cognitive decline. [102, 103] Exaggerated cardiovascular responses to stimuli in SCI, such as urinary bladder distension or postural changes, reflect impaired baroreceptor reflexes and increased sensitivity to vasopressin and noradrenaline. [104] Our findings emphasize the need for BPV and altered BP regulation monitoring in individuals living with SCI, especially for high-level injuries.

Consistent with prior research, lesion-dependent cardiovascular impairments necessitate a comprehensive assessment beyond mean 24-h BP values, incorporating BPV and nocturnal dipping patterns. [78] While 24-h ABPM is ideal, other BPV measurement methods, such as home monitoring (e.g., self-measured BP readings) and clinic measurements (e.g., standardized in-office readings), also predict cardiovascular outcomes, though these indices are not interchangeable. [34] Increased BPV independently predicts cardiovascular morbidity and mortality in the general population, [105, 106] with all BPV indices showing prognostic value, despite moderate inter-index correlations. [63,107–109] Addressing autonomic dysfunction and targeting BPV stability via tailored interventions may reduce cardiovascular risks in the SCI population, warranting further studies investigating the effect of  different therapeutic strategies. [107, 109]

### Limitations

Despite its strengths, our study has a few considerations. While data were mainly collected from a sample representative of the wider SCI population ( ~ 80% male) with the most severe cardiovascular consequences, these characteristics may limit the generalizability to females with SCI. Additionally, the data were merged from different studies, which may introduce some variability; however, a sensitivity analysis was performed to demonstrate the consistency of the findings. Moreover, our study focused on a 24-h ABPM period, which provides valuable information on diurnal BP patterns, but does not account for the longer-term variations and seasonal influences that could affect BP and autonomic function. [110] Factors such as lifestyle (e.g., physical activity levels), medication use, and psychological stressors were not measured and should be considered in future studies. Although approximately 80% of valid readings were protocol-scheduled and approximately 20% were participant-triggered, participant-triggered measurements may have inflated the dataset for symptomatic extremes. However scheduled-only sensitivity analysis remained directionally consistent with the main findings and provides some reassurance. Inconsistencies with how diary logs were completed prevented a reliable analysis of whether events clustered around activities such as transfers, bowel/bladder care, meals, exertion, or postural change. Nighttime BP was sampled less frequently than daytime BP, which may have underestimated the frequency and magnitude of nocturnal excursions. Although the hourly-thinning sensitivity analysis did not reveal any differences, the approach does not fully overcome this limitation. Finally, two ABPM devices (Mobil-O-Graph and Meditech card(X)plore) were used across sub-studies. Although both devices have been validated for brachial BP measurement according to international protocols, [40] device-level assignment per participant was not retained, so device-specific algorithmic effects cannot be completely excluded. While time-domain BPV indices (SD, CoV, ARV, VIM) are unaffected by constant calibration differences and our standardized sampling/processing reduces device-related variation differences in intrinsic measurement properties could still influence BPV values. Additionally, our between-study sensitivity analyses and conditional inference trees pointed to NLI as the primary determinant of BPV; if device type had been a dominant driver, we would expect inconsistent effects by study (which were not observed).

## Conclusion

Our findings emphasize the significant impact of SCI, particularly C-SCI, on BPV, cardiovascular regulation, and nocturnal dipping. Individuals with C-SCI exhibited greater BP instability and a higher hypotensive load during daytime, reflecting impaired sympathetic control. These alterations likely contribute to the elevated cardiovascular morbidity observed in this population. Collectively, the results highlight the necessity for targeted cardiovascular monitoring and intervention strategies in this vulnerable population. Further research is needed to explore the underlying mechanisms, clinical implications, and therapeutic approaches for managing BP dysregulation in individuals living with SCI.

## Summary

### What is known about the topic

- **SCI at or above T6 disrupts autonomic cardiovascular control**. This can produce episodic hypertension (autonomic dysreflexia) and hypotension in daily life.
- **Ambulatory BP monitoring in SCI shows disrupted circadian BP regulation**. Blunted or absent nocturnal dipping has been reported, especially in tetraplegia.
- **Higher BP variability predicts adverse cardiovascular outcomes in non-injured populations**. However, comprehensive BPV profiling across SCI injury levels remains limited.

### What this study adds

- **Cervical SCI exhibits greater 24-h systolic BP variability across complementary indices**. SD, CoV, ARV, and VIM were all higher in C-SCI than UT-SCI and non-injured controls, with neurological level of injury as the dominant predictor.
- **Nocturnal BP dipping is impaired in cervical SCI, with more reverse dipping**. This indicates substantial disruption of circadian BP regulation beyond differences in mean BP.
- **Both cervical and upper-thoracic SCI show increased susceptibility to hypotensive events**. SCI groups also demonstrated more right-skewed (often non-Gaussian) SBP distributions, consistent with greater BP lability in free-living conditions.

## Supplementary information

Supplemental File

STARD-2015-checklist

## Data Availability

The data that support the findings of this study are available from the corresponding author upon reasonable request, subject to institutional and ethical restrictions.

## References

[1] Dietz V, Harkema SJ. Locomotor activity in spinal cord-injured persons. J Appl Physiol. 2004;96:1954–60. 10.1152/japplphysiol.00942.2003 15075315

[2] Krassioukov A. Autonomic function following cervical spinal cord injury. Respir Physiol Neurobiol. 2009;169:157–64. 10.1016/j.resp.2009.08.003 19682607

[3] Taylor JA. Autonomic consequences of spinal cord injury. Auton Neurosci. 2018;209:1–3. 10.1016/J.AUTNEU.2017.09.015 28967580

[4] Krassioukov A, Claydon VE. The clinical problems in cardiovascular control following spinal cord injury: an overview. Prog Brain Res. 2006;152:223–9. 10.1016/S0079-6123(05)52014-4 16198703

[5] Myers J, Lee M, Kiratli J. Cardiovascular disease in spinal cord injury: an overview of prevalence, risk, evaluation, and management. Am J Phys Med Rehabil. 2007;86:142–52. 10.1097/PHM.0b013e31802f0247 17251696

[6] Wulf MJ, Tom VJ. Consequences of spinal cord injury on the sympathetic nervous system. Front Cell Neurosci. 2023;17:999253 10.3389/FNCEL.2023.999253 36925966 PMC10011113

[7] Krassioukov AV, Furlan JC, Fehlings MG. Autonomic dysreflexia in acute spinal cord injury: an under-recognized clinical entity. J Neurotrauma. 2003;20:707–16. 10.1089/089771503767869944 12965050

[8] Claydon VE, Hol AT, Eng JJ, Krassioukov AV. Cardiovascular responses and postexercise hypotension after arm cycling exercise in subjects with spinal cord injury. Arch Phys Med Rehabil. 2006;87:1106–14. 16876557 10.1016/j.apmr.2006.05.011

[9] Claydon VE, Steeves JD, Krassioukov A. Orthostatic hypotension following spinal cord injury: understanding clinical pathophysiology. Spinal Cord. 2006;44:341–51. 10.1038/sj.sc.3101855 16304564

[10] Hansen RM, Krogh K, Sundby J, Krassioukov A, Hagen EM. Postprandial hypotension and spinal cord injury. J Clin Med. 2021;10:1417 10.3390/JCM10071417 33915893 PMC8037943

[11] Biering-Sørensen F, Biering-Sørensen T, Liu N, Malmqvist L, Wecht JM, Krassioukov A. Alterations in cardiac autonomic control in spinal cord injury. Auton Neurosci. 2018;209:4–18. 10.1016/J.AUTNEU.2017.02.004 28228335

[12] Krassioukov AV, Weaver LC. Anatomy of the autonomic nervous system. Physical medicine and rehabilitation. 1996;10:1–4.

[13] Mancia G, Grassi G The autonomic nervous system and hypertension. Circ Res. 2014;114:1804–14. 10.1161/CIRCRESAHA.114.302524 24855203

[14] Wang S, Wecht JM, Ugiliweneza B, Legg Ditterline B. Increased prevalence of blood pressure instability over twenty-four hours in chronic spinal cord injury. Neurotrauma Rep. 2022;3:522 10.1089/NEUR.2022.0007 36479365 PMC9718427

[15] Parati G, Stergiou GS, Dolan E, Bilo G. Blood pressure variability: clinical relevance and application. The Journal of Clinical Hypertension. 2018;20:1133–7. 10.1111/JCH.13304 30003704 PMC8030809

[16] Hubli M, Gee CM, Krassioukov AV. Refined assessment of blood pressure instability after spinal cord injury. Am J Hypertens. 2015;28:173–81. 10.1093/ajh/hpu122 24990527

[17] Karlsson AK, Friberg P, Lönnroth P, Sullivan L, Elam M. Regional sympathetic function in high spinal cord injury during mental stress and autonomic dysreflexia. Brain. 1998;121:1711–9. 10.1093/BRAIN/121.9.1711 9762959

[18] Sachdeva R, Nightingale TE, Krassioukov AV. The blood pressure pendulum following spinal cord injury: implications for vascular cognitive impairment. International journal of molecular sciences. NLM (Medline). 2019;20:2464 10.3390/ijms20102464 PMC6567094 31109053

[19] Liu Y, Luo X, Jia H, Yu B. The effect of blood pressure variability on coronary atherosclerosis plaques. Front Cardiovasc Med. 2022;9:803810 10.3389/FCVM.2022.803810 35369353 PMC8965230

[20] Parati G, Lentelme P. Blood pressure variability, target organ damage and cardiovascular events. J Hypertens. 2002;20:1725–9. 12195111 10.1097/00004872-200209000-00014

[21] Rothwell PM, Howard SC, Dolan E, O’Brien E, Dobson JE, Dahlöf B, et al. Prognostic significance of visit-to-visit variability, maximum systolic blood pressure, and episodic hypertension. The Lancet. 2010;375:895–905. 10.1016/S0140-6736(10)60308-X 20226988

[22] Kikuya M, Ohkubo T, Metoki H, Asayama K, Hara A, Obara T, et al. Day-by-day variability of blood pressure and heart rate at home as a novel predictor of prognosis: the ohasama study. Hypertension. 2008;52:1045–50. 10.1161/HYPERTENSIONAHA.107.104620 18981332

[23] Yee B, Nightingale TE, Ramirez AL, Walter M, Krassioukov AV. Heart rate changes associated with autonomic dysreflexia in daily life of individuals with chronic spinal cord injury. Spinal Cord. 2022;60:1030–6. 10.1038/s41393-022-00820-y 35680988

[24] Hubli M, Krassioukov AV. Ambulatory blood pressure monitoring in spinal cord injury: clinical practicability. J Neurotrauma. 2014;31:789–97. 10.1089/neu.2013.3148 24175653 PMC3997095

[25] Munakata M, Kameyama J, Nunokawa T, Ito N, Yoshinaga K. Altered mayer wave and baroreflex profiles in high spinal cord injury. Am J Hypertens. 2001;14:141–8. 10.1016/S0895-7061(00)01236-X 11243305

[26] Peixoto AJ, White WB. Circadian blood pressure: clinical implications based on the pathophysiology of its variability. Kidney Int. 2007;71:855–60. 10.1038/SJ.KI.5002130 17377513

[27] Chadachan VM, Ye MT, Tay JC, Subramaniam K, Setia S. Understanding short-term blood-pressure-variability phenotypes: from concept to clinical practice. Int J Gen Med. 2018;11:241–54. 10.2147/IJGM.S164903 29950885 PMC6018855

[28] Parati G, Ochoa JE, Salvi P, Lombardi C, Bilo G. Prognostic value of blood pressure variability and average blood pressure levels in patients with hypertension and diabetes. Diabetes Care. 2013;36:S312 10.2337/DCS13-2043 23882065 PMC3920798

[29] Pickering TG, Hall JE, Appel LJ, Falkner BE, Graves J, Hill MN, et al. Recommendations for blood pressure measurement in humans and experimental animals: part 1: blood pressure measurement in humans: a statement for professionals from the subcommittee of professional and public education of the American heart association cou. Circulation. 2005;111:697–716. 10.1161/01.CIR.0000154900.76284.F6 15699287

[30] Kim BK, Kim YM, Lee Y, Lim YH, Shin J. A reverse dipping pattern predicts cardiovascular mortality in a clinical cohort. J Korean Med Sci. 2013;28:1468 10.3346/JKMS.2013.28.10.1468 24133351 PMC3792601

[31] Frisbie JH. Unstable baseline blood pressure in chronic tetraplegia. Spinal Cord. 2007;45:92–5. 10.1038/SJ.SC.3101920 16568144

[32] Munakata M, Kameyama J, Kanazawa M, Nunokawa T, Moriai N, Yoshinaga K. Circadian blood pressure rhythm in patients with higher and lower spinal cord injury: simultaneous evaluation of autonomic nervous activity and physical activity. J Hypertens. 1997;15:1745–9. 10.1097/00004872-199715120-00083 9488233

[33] Rosado-Rivera D, Radulovic M, Handrakis JP, Cirnigliaro CM, Jensen AM, Kirshblum S, et al. Comparison of 24-hour cardiovascular and autonomic function in paraplegia, tetraplegia, and control groups: Implications for cardiovascular risk. J Spinal Cord Med. 2011;34:395–403. 10.1179/2045772311Y.0000000019 21903013 PMC3152811

[34] Stevens SL, Wood S, Koshiaris C, Law K, Glasziou P, Stevens RJ, et al. Blood pressure variability and cardiovascular disease: systematic review and meta-analysis. BMJ. 2016;354:i4098 10.1136/BMJ.I4098 27511067 PMC4979357

[35] Walter M, Kran SL, Ramirez AL, Rapoport D, Nigro MK, Stothers L, et al. Intradetrusor onabotulinumtoxina injections ameliorate autonomic dysreflexia while improving lower urinary tract function and urinary incontinence-related quality of life in individuals with cervical and upper thoracic spinal cord injury. J Neurotrauma. 2020;37:2023–7. 10.1089/NEU.2020.7115 32631152 PMC7470218

[36] Walter M, Ramirez AL, Lee AHX, Nightingale TE, Rapoport D, Kavanagh A, et al. Fesoterodine ameliorates autonomic dysreflexia while improving lower urinary tract function and urinary incontinence-related quality of life in individuals with spinal cord injury: a prospective phase IIa study. J Neurotrauma. 2023;40:1020–5. 10.1089/NEU.2022.0333 36178342

[37] Krassioukov AV, Currie KD, Hubli M, Nightingale TE, Alrashidi AA, Ramer L, et al. Effects of exercise interventions on cardiovascular health in individuals with chronic, motor complete spinal cord injury: protocol for a randomised controlled trial [cardiovascular health/outcomes: improvements created by exercise and education in SCI (C. BMJ Open. 2019;9:e023540 10.1136/bmjopen-2018-023540 30612110 PMC6326283

[38] Kirshblum S, Waring W. Updates for the international standards for neurological classification of spinal cord injury. Phys Med Rehabil Clin N Am. 2014;25:505–17. 10.1016/j.pmr.2014.04.001 25064785

[39] Wei W, Tölle M, Zidek W, Van Der Giet M. Validation of the mobil-O-Graph: 24 h-blood pressure measurement device. Blood Press Monit. 2010;15:225–8. 10.1097/MBP.0B013E328338892F 20216407

[40] Stergiou GS, Asmar R, Myers M, Palatini P, Parati G, Shennan A, et al. Improving the accuracy of blood pressure measurement: the influence of the european society of hypertension international protocol (ESH-IP) for the validation of blood pressure measuring devices and future perspectives. J Hypertens. 2018;36:479–87. 10.1097/HJH.0000000000001635 29384984

[41] Hermida RC, Ayala DE, Fernández JR, Mojón A, Calvo C. Influence of measurement duration and frequency on ambulatory blood pressure monitoring. Revista Española de Cardiología (English Edition). 2007;60:131–8. 10.1016/S1885-5857(07)60125-X 17338878

[42] Nunn A, Brown I. Practical issues affecting the measurement and analysis of physiological data recorded remotely from individuals with spinal cord injury (SCI) during normal daily activities. Conf Proc IEEE Eng Med Biol Soc. 2006;6025–8. 10.1109/IEMBS.2006.260404 17946737

[43] Weiss A, Beloosesky Y, Koren-Morag N, Grossman A. Association between mortality and blood pressure variability in hypertensive and normotensive elders: A cohort study. The Journal of Clinical Hypertension. 2017;19:753–6. 10.1111/JCH.12996 28345291 PMC8031228

[44] Gijón-Conde T, Graciani A, López-García E, Guallar-Castillón P, García-Esquinas E, Rodríguez-Artalejo F, et al. Short-term variability and nocturnal decline in ambulatory blood pressure in normotension, white-coat hypertension, masked hypertension and sustained hypertension: a population-based study of older individuals in Spain. Hypertens Res. 2017;40:613–9. 10.1038/hr.2017.9 28179625

[45] Jinadasa SP, Mueller A, Prasad V, Subramaniam K, Heldt T, Novack V, et al. Blood pressure coefficient of variation and its association with cardiac surgical outcomes. Anesth Analg. 2018;127:832–9. 10.1213/ANE.0000000000003362 29624524

[46] Pierdomenico SD, Di Nicola M, Esposito AL, Di Mascio R, Ballone E, Lapenna D, et al. Prognostic value of different indices of blood pressure variability in hypertensive patients. Am J Hypertens. 2009;22:842–7. 10.1038/AJH.2009.103 19498342

[47] Coccina F, Pierdomenico AM, Cuccurullo C, Pierdomenico SD. Prognostic value of average real variability of systolic blood pressure in elderly treated hypertensive patients. Blood Press Monit. 2019;24:179–84. 10.1097/MBP.0000000000000381 31116150

[48] Del Giorno R, Balestra L, Heiniger PS, Gabutti L. Blood pressure variability with different measurement methods: reliability and predictors. a proof of concept cross sectional study in elderly hypertensive hospitalized patients. Medicine. 2019;98:e16347 10.1097/MD.0000000000016347 31305424 PMC6641853

[49] Dolan E, O’Brien E. Blood pressure variability: clarity for clinical practice. Hypertension. 2010;56:179–81. 10.1161/HYPERTENSIONAHA.110.154708 20606104

[50] Mena LJ, Felix VG, Melgarejo JD, Maestre GE. 24-Hour blood pressure variability assessed by average real variability: A systematic review and meta-analysis. J Am Heart Assoc. 2017;6:e006895 10.1161/JAHA.117.006895 29051214 PMC5721878

[51] Sankari A, Badr MS, Martin JL, Ayas NT, Berlowitz DJ. Impact of spinal cord injury on sleep: current perspectives. Nat Sci Sleep. 2019;11:219 10.2147/NSS.S197375 31686935 PMC6800545

[52] Gong S, Liu K, Ye R, Li J, Yang C, Chen X. Nocturnal dipping status and the association of morning blood pressure surge with subclinical target organ damage in untreated hypertensives. The Journal of Clinical Hypertension. 2019;21:1286 10.1111/JCH.13641 31376230 PMC8030332

[53] Kario K, Hoshide S, Chia YC, Buranakitjaroen P, Siddique S, Shin J, et al. Guidance on ambulatory blood pressure monitoring: A statement from the HOPE Asia Network. The Journal of Clinical Hypertension. 2021;23:411–21. 10.1111/JCH.14128 33319412 PMC8029567

[54] Krassioukov A, Warburton DE, Teasell R, Eng JJ. Spinal cord injury rehabilitation evidence research team TSR. a systematic review of the management of autonomic dysreflexia after spinal cord injury. Arch Phys Med Rehabil. 2009;90:682–95. 10.1016/j.apmr.2008.10.017 19345787 PMC3108991

[55] Krassioukov A, Eng JJ, Warburton DE, Teasell R. A systematic review of the management of orthostatic hypotension after spinal cord injury. Arch Phys Med Rehabil. 2009;90:876–85. 10.1016/j.apmr.2009.01.009 19406310 PMC3104993

[56] Katzelnick CG, Weir JP, Jones A, Galea M, Dyson-Hudson TA, Kirshblum SC, et al. Blood pressure instability in persons with SCI: evidence from a 30-day home monitoring observation. Am J Hypertens. 2019;32:938–44. 10.1093/AJH/HPZ089 31125393

[57] Benarroch EE. Physiology and pathophysiology of the autonomic nervous system. CONTINUUM Lifelong Learning in Neurology. 2020;26:12–24. 10.1212/CON.0000000000000817 31996619

[58] Krassioukov A, Biering-Sørensen F, Donovan W, Kennelly M, Kirshblum S, Krogh K, et al. International standards to document remaining autonomic function after spinal cord injury. J Spinal Cord Med. 2012;35:201–10. 10.1179/1079026812Z.00000000053 22925746 PMC3425875

[59] Krassioukov A, Linsenmeyer TA, Beck LA, Elliott S, Gorman P, Kirshblum S, et al. Evaluation and management of autonomic dysreflexia and other autonomic dysfunctions: preventing the highs and lows: management of blood pressure, sweating, and temperature dysfunction. Top Spinal Cord Inj Rehabil. 2021;27:225 10.46292/SCI2702-225 34108837 PMC8152175

[60] Lakens D. Calculating and reporting effect sizes to facilitate cumulative science: a practical primer for t-tests and ANOVAs. Front Psychol. 2013;4:863 10.3389/FPSYG.2013.00863 24324449 PMC3840331

[61] Vincent WJ, Weir JP Statistics in kinesiology. 4th ed. Champaign, IL: Human Kinetics; 2012. 378

[62] Tanadini LG, Steeves JD, Hothorn T, Abel R, Maier D, Schubert M, et al. Identifying homogeneous subgroups in neurological disorders: unbiased recursive partitioning in cervical complete spinal cord injury. Neurorehabil Neural Repair. 2014;28:507–15. 24477680 10.1177/1545968313520413

[63] Schutte AE, Kollias A, Stergiou GS. Blood pressure and its variability: classic and novel measurement techniques. Nat Rev Cardiol. 2022;19:643 10.1038/S41569-022-00690-0 35440738 PMC9017082

[64] Veloudi P, Sharman JE. Methodological factors affecting quantification of blood pressure variability: A scoping review. J Hypertens. 2018;36:711–9. 10.1097/HJH.0000000000001606 29176390

[65] Yu J, Song Q, Bai J, Wu S, Bu P, Li Y, et al. Visit-to-visit blood pressure variability and cardiovascular outcomes in patients receiving intensive versus standard blood pressure control: insights from the STEP trial. Hypertension. 2023;80:1507–16. 10.1161/HYPERTENSIONAHA.122.20376 37170806

[66] Zhang Y, Agnoletti D, Blacher J, Safar ME. Blood pressure variability in relation to autonomic nervous system dysregulation: the X-CELLENT study. Hypertens Res. 2012;35:399–403. 10.1038/hr.2011.203 22129516

[67] Ebrahimi H, Maroufi SF, Abdollahzadegan S, Rahimi-Movaghar V. Clinical practice guideline development for autonomic dysreflexia in spinal cord injury. Med J Islam Repub Iran. 2023;37:109 10.47176/MJIRI.37.109 38145189 PMC10744198

[68] Barry W, St Andre JR, Evans CT, Sabharwal S, Miskevics S, Weaver FM, et al. Hypertension and antihypertensive treatment in veterans with spinal cord injury and disorders. Spinal Cord. 2013;51:109–15. 10.1038/sc.2012.122 23147130

[69] Li HL, Lin HJ, Muo CH, Lu CY, Kuo CC, Chen PC. Blood Pressure variability and risk of cardiovascular events and mortality in real-world clinical settings. J Am Heart Assoc. 2025;14:37658. 10.1161/JAHA.124.037658 PMC12229178 40417802

[70] Ebinger JE, Driver M, Ouyang D, Botting P, Ji H, Rashid MA, et al. Variability independent of mean blood pressure as a real-world measure of cardiovascular risk. EClinicalMedicine. 2022;48:101442 10.1016/j.eclinm.2022.101442 35706499 PMC9112125

[71] Bauman WA, Spungen AM. Disorders of carbohydrate and lipid metabolism in veterans with paraplegia or quadriplegia: A model of premature aging. Metabolism. 1994;43:749–56. 10.1016/0026-0495(94)90126-0 8201966

[72] Benitez-Albiter A, Anderson CP, Jones M, Park SS, Layec G, Park SY. Contributing factors to endothelial dysfunction in individuals with spinal cord injuries. Pulse (Basel). 2024;12:49–57. 10.1159/000539199 39022560 PMC11250044

[73] Phillips AA, Cote AT, Bredin SSD, Krassioukov AV, Warburton DER. Aortic stiffness increased in spinal cord injury when matched for physical activity. Med Sci Sports Exerc. 2012;44:2065–70. 10.1249/MSS.0b013e3182632585 22688833

[74] Phillips AA, Cote AT, Warburton DER. A systematic review of exercise as a therapeutic intervention to improve arterial function in persons living with spinal cord injury. Spinal Cord. 2011;49:702–14. 10.1038/sc.2010.193 21339761

[75] Currie KD, Hubli M, MacDonald MJ, Krassioukov AV. Associations between arterial stiffness and blood pressure fluctuations after spinal cord injury. Spinal Cord. 2019;57:1057–63. 10.1038/s41393-019-0316-y 31217517

[76] Bilo G, Parati G. Blood pressure variability and kidney disease: another vicious circle? J Hypertens. 2018;36:1019–21. 10.1097/HJH.0000000000001707 29578957

[77] Miyatani M, Szeto M, Moore C, Oh PI, McGillivray CF, Craven BC. Exploring the associations between arterial stiffness and spinal cord impairment: A cross-sectional study. J Spinal Cord Med. 2014;37:556–64. 10.1179/2045772314Y.0000000261 25229737 PMC4166190

[78] West CR, Mills P, Krassioukov AV. Influence of the neurological level of spinal cord injury on cardiovascular outcomes in humans: a meta-analysis. Spinal Cord. 2012;50:484–92. 10.1038/sc.2012.17 22391687

[79] Balthazaar SJT, Alrashidi AA, Currie KD, Hubli M, Hodgkiss DD, Nightingale TE, et al. Effects of active upper-limb and passive lower-limb exercise on 24-hour ambulatory blood pressure outcomes in individuals with motor-complete spinal cord injury: A case-series. Journal of Spinal Cord Medicine. 2026;12:1–11. 10.1080/10790268.2026.2630501 PMC13591975 42117700

[80] Gavriilaki M, Anyfanti P, Nikolaidou B, Lazaridis A, Gavriilaki E, Douma S, et al. Nighttime dipping status and risk of cardiovascular events in patients with untreated hypertension: A systematic review and meta‐analysis. The Journal of Clinical Hypertension. 2020;22:1951 10.1111/JCH.14039 33164307 PMC8030020

[81] Goh MY, Millard MS, Wong ECK, Berlowitz DJ, Graco M, Schembri RM, et al. Comparison of diurnal blood pressure and urine production between people with and without chronic spinal cord injury. Spinal Cord. 2018;56:847–55. 10.1038/s41393-018-0081-3 29500404

[82] Yano Y, Kario K. Nocturnal blood pressure and cardiovascular disease: a review of recent advances. Hypertens Res. 2012;35:695–701. 10.1038/hr.2012.26 22378470

[83] Bouhanick B, Bongard V, Amar J, Bousquel S, Chamontin B. Prognostic value of nocturnal blood pressure and reverse-dipping status on the occurrence of cardiovascular events in hypertensive diabetic patients. Diabetes Metab. 2008;34:560–7. 10.1016/J.DIABET.2008.05.005 18926758

[84] Minutolo R, Agarwal R, Borrelli S, Chiodini P, Bellizzi V, Nappi F, et al. Prognostic role of ambulatory blood pressure measurement in patients with nondialysis chronic kidney disease. Arch Intern Med. 2011;171:1090–8. 10.1001/ARCHINTERNMED.2011.230 21709109

[85] Shin J, Kline S, Moore M, Gong Y, Bhanderi V, Schmalfuss CM, et al. Association of diurnal blood pressure pattern with risk of hospitalization or death in men with heart failure. J Card Fail. 2007;13:656–62. 10.1016/J.CARDFAIL.2007.04.013 17923358 PMC2655230

[86] Komori T, Eguchi K, Saito T, Nishimura Y, Hoshide S, Kario K. Riser blood pressure pattern is associated with mild cognitive impairment in heart failure patients. Am J Hypertens. 2016;29:194–201. 10.1093/AJH/HPV086 26066331

[87] García-Ortiz L, Gómez-Marcos MA, Martín-Moreiras J, González-Elena LJ, Recio-Rodriguez JI, Castaño-Sánchez Y, et al. Pulse pressure and nocturnal fall in blood pressure are predictors of vascular, cardiac and renal target organ damage in hypertensive patients (LOD-RISK study). Blood Press Monit. 2009;14:145–51. 10.1097/MBP.0B013E32832E062F 19581802

[88] Pogue V, Rahman M, Lipkowitz M, Toto R, Miller E, Faulkner M, et al. Disparate estimates of hypertension control from ambulatory and clinic blood pressure measurements in hypertensive kidney disease. Hypertension. 2009;53:20–7. 10.1161/HYPERTENSIONAHA.108.115154 19047584

[89] Cuspidi C, Sala C, Tadic M, Gherbesi E, De Giorgi A, Grassi G, et al. Clinical and prognostic significance of a reverse dipping pattern on ambulatory monitoring: An updated review. The Journal of Clinical Hypertension. 2017;19:713–21. 10.1111/JCH.13023 28692165 PMC8031119

[90] Wecht JM, La Fountaine MF, Handrakis JP, West CR, Phillips A, Ditor DS, et al. Autonomic nervous system dysfunction following spinal cord injury: cardiovascular, cerebrovascular, and thermoregulatory effects. Curr Phys Med Rehabil Rep. 2015;3:197–205. 10.1007/s40141-015-0093-2

[91] Sweis R, Biller J. Systemic complications of spinal cord injury. Curr Neurol Neurosci Rep. 2017;17:1–8. 10.1007/S11910-017-0715-4 28188542

[92] Lucas SJE, Tzeng YC, Galvin SD, Thomas KN, Ogoh S, Ainslie PN. Influence of changes in blood pressure on cerebral perfusion and oxygenation. Hypertension. 2010;55:698–705. 10.1161/HYPERTENSIONAHA.109.146290 20083726

[93] Kim HL. Arterial stiffness and hypertension. Clin Hypertens. 2023;29:31 10.1186/S40885-023-00258-1 38037153 PMC10691097

[94] Mahmood SS, Levy D, Vasan RS, Wang TJ. The framingham heart study and the epidemiology of cardiovascular diseases: a historical perspective. Lancet. 2014;383:999 10.1016/S0140-6736(13)61752-3 24084292 PMC4159698

[95] McCarthy CP, Natarajan P. Systolic Blood Pressure and Cardiovascular Risk: Straightening the Evidence. Hypertension. 2023;80:577–9. 10.1161/HYPERTENSIONAHA.123.20788 36791225 PMC9942105

[96] West CR, AlYahya A, Laher I, Krassioukov A. Peripheral vascular function in spinal cord injury: a systematic review. Spinal Cord. 2013;51:10–9. 10.1038/sc.2012.136 23184028

[97] Tochikubo O, Hishiki S, Miyajima E, Ishii M. Statistical base value of 24-hour blood pressure distribution in patients with essential hypertension. Hypertension. 1998;32:430–6. 10.1161/01.HYP.32.3.430 9740607

[98] Kario K, Schwartz JE, Pickering TG. Ambulatory physical activity as a determinant of diurnal blood pressure variation. Hypertension. 1999;34:685–91. 10.1161/01.HYP.34.4.685 10523347

[99] Kumar A, Hung OY, Piccinelli M, Eshtehardi P, Corban MT, Sternheim D, et al. Low coronary wall shear stress is associated with severe endothelial dysfunction in patients with nonobstructive coronary artery disease. JACC Cardiovasc Interv. 2018;11:2072–80. 10.1016/J.JCIN.2018.07.004 30268874 PMC6217963

[100] Boon R, Horrevoets A. Key transcriptional regulators of the vasoprotective effects of shear stress. Hamostaseologie. 2009;29:39–40. 19151844

[101] Kario K, Shimada K, Pickering TG. Clinical implication of morning blood pressure surge in hypertension. J Cardiovasc Pharmacol. 2003;42:S87–91. 10.1097/00005344-200312001-00019 14871036

[102] Oishi E, Ohara T, Sakata S, Fukuhara M, Hata J, Yoshida D, et al. Day-to-day blood pressure variability and risk of dementia in a general japanese elderly population: the hisayama study. Circulation. 2017;136:516–25. 10.1161/CIRCULATIONAHA.116.025667 28784822 PMC5548511

[103] Mitchell GF, Van Buchem MA, Sigurdsson S, Gotal JD, Jonsdottir MK, Kjartansson Ó, et al. Arterial stiffness, pressure and flow pulsatility and brain structure and function: the age, gene/environment susceptibility-reykjavik study. Brain. 2011;134:3398–407. 10.1093/BRAIN/AWR253 22075523 PMC3212721

[104] Krum H, Howes LG, Brown DJ, Louis WJ. Blood pressure variability in tetraplegic patients with autonomic hyperreflexia. Paraplegia. 1989;27:284–8. 10.1038/SC.1989.42 2780084

[105] Hitzig SL, Eng JJ, Miller WC, Sakakibara BM. An evidence-based review of aging of the body systems following spinal cord injury. Spinal Cord. 2011;49:684–701. 10.1038/sc.2010.178 21151191 PMC3181216

[106] Palatini P, Reboldi G, Beilin LJ, Casiglia E, Eguchi K, Imai Y, et al. Added predictive value of night-time blood pressure variability for cardiovascular events and mortality: the ambulatory blood pressure-international study. Hypertension. 2014;64:487–93. 10.1161/HYPERTENSIONAHA.114.03694 24935939

[107] Mena LJ, Investigators the ID on ABP in R to CO (IDACO), Maestre GE, Investigators the ID on ABP in R to CO (IDACO), Hansen TW, Investigators the ID on ABP in R to CO (IDACO). et al. How many measurements are needed to estimate blood pressure variability without loss of prognostic information? Am J Hypertens. 2014;27:46–55. 10.1093/AJH/HPT142 23955605 PMC3848629

[108] Boubouchairopoulou N, Ntineri A, Kollias A, Destounis A, Stergiou GS. Blood pressure variability assessed by office, home, and ambulatory measurements: comparison, agreement, and determinants. Hypertens Res. 2021;44:1617–24. 10.1038/S41440-021-00736-9 34599293

[109] Juhanoja EP, Niiranen TJ, Johansson JK, Puukka PJ, Jula AM. Agreement between ambulatory, home, and office blood pressure variability. J Hypertens. 2016;34:61–7. 10.1097/HJH.0000000000000772 26630214

[110] Stergiou GS, Myrsilidi A, Kollias A, Destounis A, Roussias L, Kalogeropoulos P. Seasonal variation in meteorological parameters and office, ambulatory and home blood pressure: predicting factors and clinical implications. Hypertens Res. 2015;38:869–75. 10.1038/hr.2015.96 26333360

