# Supplementary material for: Blood pressure variability in daily life: comparing individuals with cervical and upper-thoracic spinal cord injury to non-injured controls
Source: J Hum Hypertens. 2026 Jul 9;40(9):712–23. doi: 10.1038/s41371-026-01179-w (PMC13558047; doi:10.1038/s41371-026-01179-w)
Supplement: Supplementary file 1 [file 41371_2026_1179_MOESM1_ESM.pdf]

Supplementary Table 1. Summary of Characteristics from the Clinical Trials and Observational Studies

	NCT02298660	NCT02676154	NCT01718977	H16-01458
**Study Design**	Prospective, open-label, pre/post comparison study	Phase II, open-label, exploratory, non-blinded, non-randomized, single-centre study	Prospective, multi-centre, randomized controlled clinical trial	Cross-sectional comparison study
**Study Duration**	April 2013 to December 2017	April 2016 to January 2019	April 2013 to April 2019	February 2018 to August 2023
**Participants**	Males and females, 18-65 years, chronic traumatic SCI at or above T6, with AD and NDO	Males and females with chronic traumatic SCI at or above T6 with documented AD triggered by NDO	Males and females, 18-65 years, chronic traumatic SCI at or above T6	Males and females, 19-65 years, chronic traumatic SCI at or above T6. Age-matched non-injured controls
**Inclusion Criteria**	Chronic, traumatic SCI >1 year, AD during UDS, NDO, capable of CIC, resistant to anticholinergics	Chronic SCI at or above T6, documented AD triggered by NDO	Chronic, motor-complete, traumatic SCI >1 year with NLI C4-T6	Chronic, motor complete, traumatic SCI > 1 year with NLI C4 – T6, with a documented presence of AD
**Exclusion Criteria**	TBI, previous bladder Botox use, genitourinary disease/surgery, multiple injury levels, acute UTI, cardiovascular disease	Previous botulinum toxin (9 months washout), recent antimuscarinics (3 weeks washout)	Any active or history of medical issues (e.g. cardiovascular or cardiopulmonary problems/disease) at enrolment	History of untreated cardiovascular disease, type 1 diabetes, untreated type 2 diabetes, obesity, severe brain injury, untreated depression
**Interventions**	Botox injections post baseline evaluations	Fesoterodine (4-8 mg) administered for 12 weeks	Exercise training with baseline and follow-up assessments	N/A
**Primary Outcome**	Change in AD severity measured by ΔSBP during CIC and ABPM	Effect of fesoterodine in reducing AD severity during UDS and daily life	24-hour BP monitoring for BP lability, AD, and OH symptoms	Cerebrovascular health (e.g., MR derived cerebrovascular reactivity and cognition)
**Secondary Outcomes**	Daytime and nighttime BP changes, ΔHR during AD episodes	Frequency and severity of AD, QoL improvement (AD HR-QoL), bladder incontinence (I-QoL), cognitive function (MoCA), bowel function (Bristol Stool Scale, NBD), UDS improvements	AD, OH, and subjective symptoms assessed with autonomic questionnaire	24-hour BP monitoring for BP lability, AD, and OH symptoms
Summary of study characteristics from clinical trials and observational studies in individuals with traumatic spinal cord injury (SCI) at or above T6. Details include study identifiers, design, duration, participant characteristics, inclusion/exclusion criteria, interventions, and primary and secondary outcomes. Abbreviations: SCI, spinal cord injury; AD, autonomic dysreflexia; NDO, neurogenic detrusor overactivity; CIC, clean intermittent catheterization; UDS,				

urodynamic study; QoL, quality of life; HR-QoL, health-related quality of life; MoCA, Montreal Cognitive Assessment; NBD, neurogenic bowel dysfunction; ΔSBP, change in systolic blood pressure; ΔHR, change in heart rate; ABPM, ambulatory blood pressure monitoring; OH, orthostatic hypotension.

Supplementary Table 2. Group Comparisons of 24-Hour Ambulatory Blood Pressure and Heart Rate Measures

	**Cervical SCI** Mean ± SD (95% CI)	**Upper-Thoracic SCI** Mean ± SD (95% CI)	**Non-Injured Controls** Mean ± SD (95% CI)	**One-way ANOVA or Kruskal-Wallis** (*P* value)	**Effect size** (Eta-squared; η^2)	**Between-group Comparisons** (*P* value)
**Median BP**						
Median SBP 24h (mmHg)	109 ± 13 (105 to 113)	115 ± 12 (110 to 121)	127 ± 10 (122 to 132)	**<0.001**	0.268**	C-SCI and UT-SCI: 0.178<br>**UT-SCI and NI-C: 0.012**<br>**C-SCI and NI-C: <0.001**
Median SBP ☼ (mmHg)	109 ± 13 (105 to 113)	117 ± 13 (111 to 122)	129 ± 9 (125 to 134)	**<0.001**	0.293**	C-SCI and UT-SCI: 0.089<br>**UT-SCI and NI-C: 0.011**<br>**C-SCI and NI-C: <0.001**
Median SBP ☽ (mmHg)	107 ± 11 (104 to 111)	102 ± 11 (97 to 107)	109 ± 10 (105 to 114)	0.112	0.055	C-SCI and UT-SCI: 0.250<br>UT-SCI and NI-C: 0.150<br>C-SCI and NI-C: >0.999
Median DBP 24h (mmHg)	65 ± 9 (62 to 67)	67 ± 7 (64 to 70)	78 ± 10 (73 to 83)	**<0.001**	0.259**	C-SCI and UT-SCI: 0.968<br>**UT-SCI and NI-C: 0.002**<br>**C-SCI and NI-C: <0.001**
Median DBP ☼ (mmHg)	65 ± 10 (62 to 68)	68 ± 7 (65 to 72)	79 ± 10 (75 to 84)	**<0.001**	0.272**	C-SCI and UT-SCI: 0.653<br>**UT-SCI and NI-C: 0.002**<br>**C-SCI and NI-C: <0.001**
Median DBP ☽ (mmHg)	61 ± 10 (58 to 64)	56 ± 9 (52 to 60)	62 ± 8 (58 to 66)	0.107	0.056	C-SCI and UT-SCI: 0.200<br>UT-SCI and NI-C: 0.170<br>C-SCI and NI-C: >0.999
**Nocturnal Dip**						
Median SBP (%)	6 ± 12 (3 to 10)	17 ± 8 (13 to 20)	18 ± 7 (15 to 21)	**<0.001**	0.221**	**C-SCI and UT-SCI: 0.002**<br>UT-SCI and NI-C: >0.999<br>**C-SCI and NI-C: 0.001**
Median DBP (%)	10 ± 14 (6 to 14)	23 ± 11 (18 to 28)	26 ± 6 (23 to 29)	**<0.001**	0.262**	**C-SCI and UT-SCI: 0.001**<br>UT-SCI and NI-C: >0.999<br>**C-SCI and NI-C: <0.001**
**Heart Rate**						
Median HR 24h (bpm)	68 ± 10 (65 to 71)	74 ± 11 (70 to 79)	66 ± 12 (60 to 72)	**0.048**	0.076*	C-SCI and UT-SCI: 0.119<br>UT-SCI and NI-C: 0.076<br>C-SCI and NI-C: >0.999
Median HR ☼ (bpm)	70 ± 10 (66 to 73)	76 ± 11 (71 to 81)	67 ± 12 (62 to 73)	0.053	0.074*	C-SCI and UT-SCI: 0.119<br>UT-SCI and NI-C: 0.076

	**Cervical SCI** Mean ± SD (95% CI)	**Upper-Thoracic SCI** Mean ± SD (95% CI)	**Non-Injured Controls** Mean ± SD (95% CI)	**One-way ANOVA or Kruksal-Wallis** (*P* value)	**Effect size** (Eta-squared; η^2)	**Between-group Comparisons** (*P* value)
						C-SCI and NI-C: >0.999
Median HR ☽ (bpm)	65 ± 10 (62 to 68)	66 ± 12 (61 to 72)	57 ± 14 (51 to 64)	**0.047**	0.076*	C-SCI and UT-SCI: >0.999<br>UT-SCI and NI-C: 0.069<br>C-SCI and NI-C:0.091
**Blood Pressure Variability Metrics**						
SD SBP 24h (mmHg)	18 ± 5 (16 to 19)	14 ± 4 (12 to 16)	14 ± 3 (13 to 15)	**0.004**	0.130*	**C-SCI and UT-SCI: 0.024**<br>UT-SCI and NI-C: >0.999<br>**C-SCI and NI-C: 0.023**
SD SBP ☼ (mmHg)	18 ± 6 (16 to 20)	13 ± 4 (11 to 15)	12 ± 3 (11 to 14)	**<0.001**	0.202**	**C-SCI and UT-SCI: 0.003**<br>UT-SCI and NI-C: >0.999<br>**C-SCI and NI-C: 0.002**
SD SBP ☽ (mmHg)	13 ± 6 (11 to 15)	11 ± 4 (9 to 12)	10 ± 3 (9 to 12)	0.101	0.059	C-SCI and UT-SCI: 0.320<br>UT-SCI and NI-C: >0.999<br>C-SCI and NI-C: 0.210
SD DBP 24h (mmHg)	12 ± 4 (11 to 13)	11 ± 2 (10 to 12)	12 ± 2 (10 to 13)	0.426	0.032	C-SCI and UT-SCI: 0.630<br>UT-SCI and NI-C: >0.999<br>C-SCI and NI-C: >0.999
SD DBP ☼ (mmHg)	12 ± 4 (11 to 14)	10 ± 3 (9 to 12)	10 ± 2 (9 to 11)	0.061	0.090*	C-SCI and UT-SCI: 0.150<br>UT-SCI and NI-C: >0.999<br>C-SCI and NI-C: 0.210
SD DBP ☽ (mmHg)	10 ± 4 (8 to 11)	8 ± 3 (7 to 9)	8 ± 3 (7 to 9)	0.085	0.063*	C-SCI and UT-SCI: 0.210<br>UT-SCI and NI-C: >0.999<br>C-SCI and NI-C: 0.240
CoV SBP 24h (%)	16 ± 5 (14 to 17)	12 ± 3 (11 to 14)	11 ± 2 (10 to 12)	**<0.001**	0.227**	**C-SCI and UT-SCI: 0.003**<br>UT-SCI and NI-C: >0.999<br>**C-SCI and NI-C: <0.001**
CoV SBP ☼ (%)	16 ± 5 (14 to 18)	11 ± 3 (10 to 12)	10 ± 2 (8 to 11)	**<0.001**	0.306**	**C-SCI and UT-SCI: <0.001**<br>UT-SCI and NI-C: >0.999<br>**C-SCI and NI-C: <0.001**
CoV SBP ☽ (%)	12 ± 5 (10 to 13)	10 ± 3 (9 to 12)	10 ± 3 (8 to 11)	0.0143	0.050	C-SCI and UT-SCI: 0.680<br>UT-SCI and NI-C: >0.999<br>C-SCI and NI-C: 0.200
CoV DBP 24h (%)	19 ± 6 (17 to 21)	17 ± 4 (15 to 19)	15 ± 2 (14 to 16)	**0.042**	0.106*	C-SCI and UT-SCI: 0.654<br>UT-SCI and NI-C: 0.686

	**Cervical SCI** Mean ± SD (95% CI)	**Upper-Thoracic SCI** Mean ± SD (95% CI)	**Non-Injured Controls** Mean ± SD (95% CI)	**One-way ANOVA or Kruksal-Wallis** (*P* value)	**Effect size** (Eta-squared; η^2)	**Between-group Comparisons** (*P* value)
						C-SCI and NI-C: 0.049
CoV DBP ☼ (%)	19 ± 6 (17 to 21)	15 ± 4 (13 to 17)	13 ± 3 (11 to 14)	**<0.001**	0.196**	C-SCI and UT-SCI: 0.077<br>UT-SCI and NI-C: 0.137<br>**C-SCI and NI-C: 0.001**
CoV DBP ☽ (%)	15 ± 6 (13 to 17)	14 ± 5 (12 to 16)	13 ± 4 (11 to 15)	0.263	0.035	C-SCI and UT-SCI: 0.880<br>UT-SCI and NI-C: >0.999<br>C-SCI and NI-C: 0.400
ARV SBP 24h (mmHg)	13 ± 4 (12 to 14)	11 ± 2 (10 to 12)	10 ± 2 (9 to 11)	**<0.001**	0.209**	**C-SCI and UT-SCI: 0.019**<br>UT-SCI and NI-C: 0.782<br>**C-SCI and NI-C: 0.002**
ARV SBP ☼ (mmHg)	14 ± 4 (12 to 15)	11 ± 3 (9 to 12)	10 ± 2 (9 to 11)	**<0.001**	0.224**	**C-SCI and UT-SCI: 0.004**<br>UT-SCI and NI-C: >0.999<br>**C-SCI and NI-C: 0.003**
ARV SBP ☽ (mmHg)	11 ± 5 (10 to 13)	9 ± 3 (8 to 11)	9 ± 3 (8 to 11)	0.134	0.051	C-SCI and UT-SCI: 0.270<br>UT-SCI and NI-C: >0.999<br>C-SCI and NI-C: 0.390
ARV DBP 24h (mmHg)	9 ± 2 (8 to 10)	8 ± 2 (7 to 9)	8 ± 2 (7 to 9)	0.094	0.060*	C-SCI and UT-SCI: 0.190<br>UT-SCI and NI-C: >0.999<br>C-SCI and NI-C: 0.320
ARV DBP ☼ (mmHg)	9 ± 3 (9 to 10)	8 ± 2 (7 to 9)	8 ± 2 (7 to 9)	**0.041**	0.080*	C-SCI and UT-SCI: 0.110<br>UT-SCI and NI-C: >0.999<br>C-SCI and NI-C: 0.140
ARV DBP ☽ (mmHg)	8 ± 3 (7 to 9)	6 ± 2 (6 to 7)	7 ± 2 (6 to 8)	0.148	0.049	C-SCI and UT-SCI: 0.200<br>UT-SCI and NI-C: >0.999<br>C-SCI and NI-C: 0.700
VIM SBP 24h (mmHg)	17 ± 5 (16 to 19)	14 ± 4 (12 to 15)	13 ± 2 (12 to 14)	**0.002**	0.154**	**C-SCI and UT-SCI: 0.014**<br>UT-SCI and NI-C: >0.999<br>**C-SCI and NI-C: 0.009**
VIM SBP ☼ (mmHg)	18 ± 6 (16 to 20)	13 ± 4 (11 to 15)	13 ± 3 (11 to 14)	**<0.001**	0.191**	**C-SCI and UT-SCI: 0.004**<br>UT-SCI and NI-C: >0.999<br>**C-SCI and NI-C: 0.002**
VIM SBP ☽ (mmHg)	12 ± 5 (10 to 13)	10 ± 3 (9 to 12)	9 ± 3 (8 to 11)	0.155	0.048	C-SCI and UT-SCI: 0.990<br>UT-SCI and NI-C: >0.999<br>C-SCI and NI-C: 0.180

	**Cervical SCI** Mean ± SD (95% CI)	**Upper-Thoracic SCI** Mean ± SD (95% CI)	**Non-Injured Controls** Mean ± SD (95% CI)	**One-way ANOVA or Kruskal-Wallis** (*P* value)	**Effect size** (Eta-squared; η²)	**Between-group Comparisons** (*P* value)
VIM DBP 24h (mmHg)	14 ± 4 (13 to 15)	13 ± 3 (12 to 14)	13 ± 2 (12 to 14)	0.3167	0.048	C-SCI and UT-SCI: 0.840<br>UT-SCI and NI-C: >0.999<br>C-SCI and NI-C: 0.560
VIM DBP ☼ (mmHg)	18 ± 6 (16 to 20)	14 ± 4 (12 to 16)	12 ± 3 (11 to 13)	**<0.001**	0.198**	C-SCI and UT-SCI: 0.250<br>UT-SCI and NI-C: 0.654<br>**C-SCI and NI-C: <0.001**
VIM DBP ☽ (mmHg)	13 ± 5 (11 to 14)	11 ± 4 (9 to 13)	11 ± 3 (9 to 12)	0.195	0.042	C-SCI and UT-SCI: 0.590<br>UT-SCI and NI-C: >0.999<br>C-SCI and NI-C: 0.560
**Daily Blood Pressure Events**						
Number of Hypotensive	17 ± 15 (13 to 22)	8 ± 11 (3 to 13)	1 ± 1 (0 to 1)	**<0.001**	0.224**	**C-SCI and UT-SCI: 0.018**<br>**UT-SCI and NI-C: 0.005**<br>**C-SCI and NI-C: <0.001**
Percent Hypotensive (%)	31 ± 23 (24 to 38)	13 ± 17 (6 to 21)	2 ± 3 (0 to 3)	**<0.001**	0.296**	**C-SCI and UT-SCI: 0.010**<br>**UT-SCI and NI-C: 0.008**<br>**C-SCI and NI-C: <0.001**
Number of Hypertensive	4 ± 5 (2 to 5)	3 ± 7 (0 to 6)	3 ± 5 (1 to 6)	0.265	0.005	C-SCI and UT-SCI: 0.360<br>UT-SCI and NI-C: 0.620<br>C-SCI and NI-C: >0.999
Percent Hypertensive (%)	5 ± 7 (3 to 8)	4 ± 9 (0 to 8)	6 ± 9 (2 to 10)	0.261	0.005	C-SCI and UT-SCI: 0.340<br>UT-SCI and NI-C: 0.660<br>C-SCI and NI-C: >0.999

24h, over a 24-hour period; ☼, daytime; ☽, nighttime; AD, autonomic dysreflexia; ARV, average real variability; CoV, coefficient of variation; DBP, diastolic blood pressure; SBP, systolic blood pressure; SD, standard deviation.

Bold Text – Significant Comparison ($P \leq 0.05$)

* Eta Squared value indicates a medium effect for the effect of the exercise intervention on the dependent variable ($\eta^2 \geq 0.06$)

** Eta Squared value indicates a large effect for the effect of the exercise intervention on the dependent variable ($\eta^2 \geq 0.14$)

Supplementary Table 3

Daytime systolic blood pressure data were resampled to hourly intervals using the median value within each hour to match the lower sampling frequency of nighttime data. This approach ensured comparable data density across day and night periods and allowed direct comparison of blood pressure variability (BPV) metrics between full 24-hour and hourly-thinned datasets.

**BPV Metric**	**C-SCI**	**UT-SCI**	**NI-C**	**Kruskal-Wallis H**	***P* value**	**Significant Pairwise Comparisons (Bonferroni adjusted)**
SD (mmHg)	13.5 ± 5.5	9.7 ± 3.7	9.2 ± 1.8	10.24	0.006	C-SCI > UT-SCI (*P* = 0.0437);<br>C-SCI > NI-C (*P* = 0.0229)
CoV (%)	14.7 ± 6.4	10.9 ± 3.3	7.6 ± 2.0	17.88	<0.0001	C-SCI > UT-SCI (*P* = 0.00017);<br>C-SCI > NI-C (*P* = 0.0011)
ARV (mmHg)	12.4 ± 4.0	9.0 ± 2.6	7.7 ± 2.3	13.12	0.0014	C-SCI > UT-SCI (*P* = 0.0101);<br>C-SCI > NI-C (*P* = 0.0129)
VIM (mmHg)	13.9 ± 5.2	10.6 ± 3.9	9.6 ± 1.7	9.41	0.0091	C-SCI > UT-SCI (*P* = 0.0497);

Supplementary Figure 1

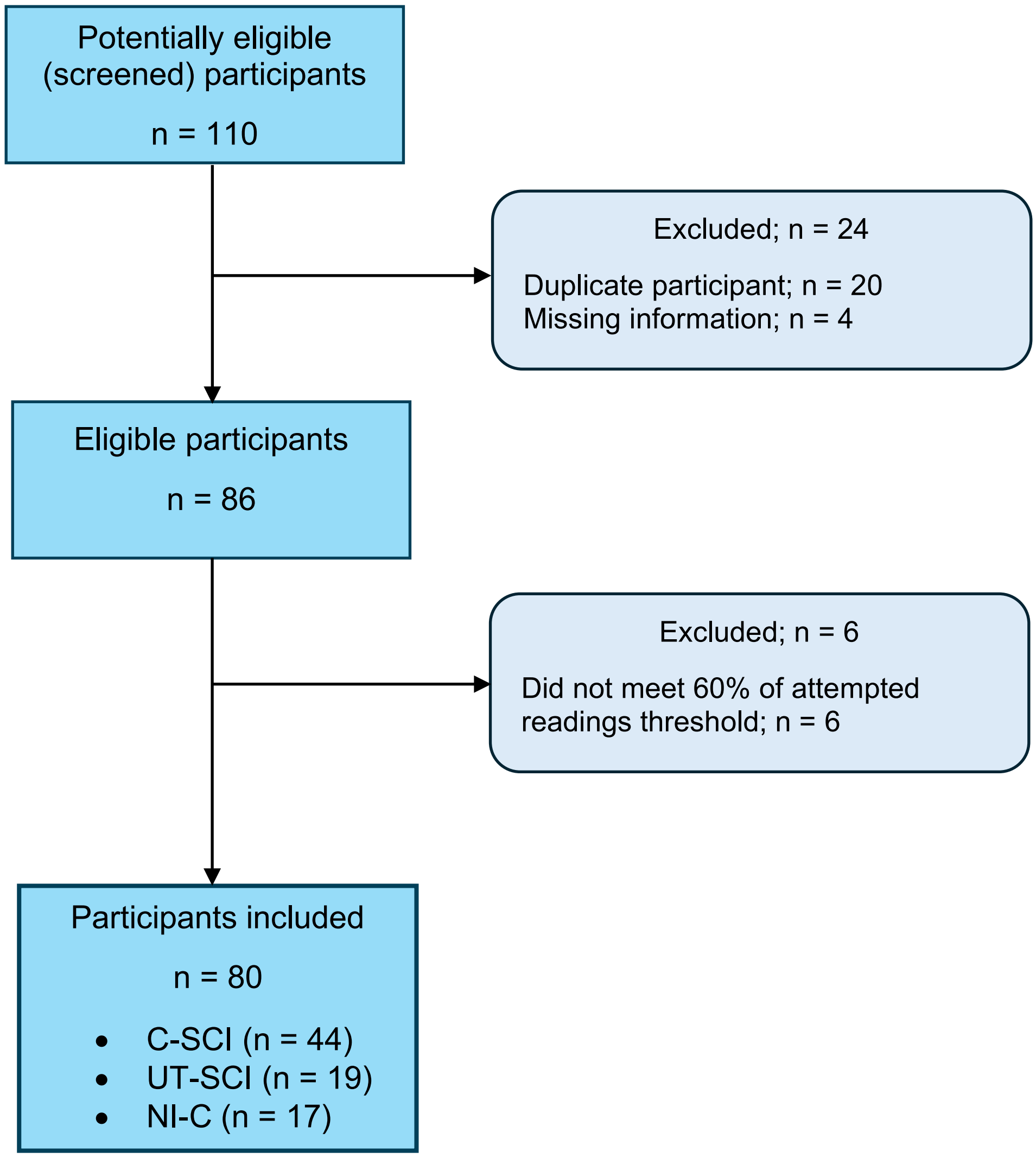

Supplementary Figure 1. STARD diagram to report flow of participants in the study.
The flowchart shows the number of participants at each stage of the study, including those assessed for eligibility, those excluded from the study, and the final number of participants in the analysis. The exclusion criteria were provided with reasons for exclusion to understand the potential sources of bias.

Supplementary Figure 2

There was a higher prevalence of non-dippers (SBP and DBP nocturnal dip decrease by 0-10%) and reverse dippers (SBP and DBP nocturnal dip increase) for individuals with cervical spinal cord injury (C-SCI). Dippers (SBP nocturnal dip decrease by 10-20%) were more prevalent in the upper-thoracic spinal cord injury (UT-SCI) and non-injured control (NI-C) groups. Though extreme dippers (SBP and DBP nocturnal dip decrease by ≥20%) were most prevalent in the NI-C group, the young age of the participant cohort would suggest this does not carry a greater cardiovascular risk compared to an older population [113].

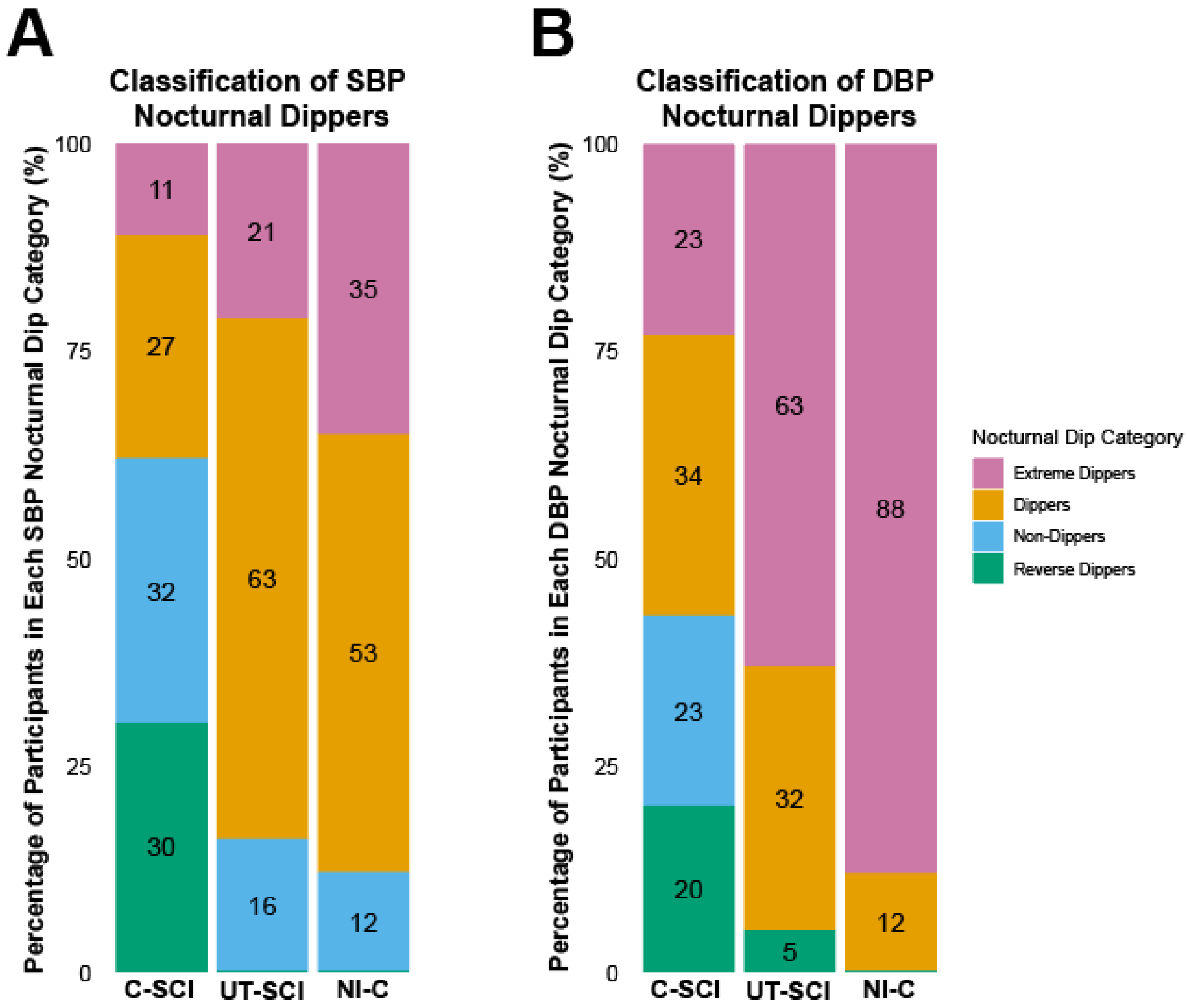

Supplementary Figure 2. Stacked bar charts of systolic and diastolic nocturnal dip categories. The proportional distribution of individuals within each category of nocturnal dip for each group are displayed. **(A) *Classification of SBP Nocturnal Dippers:*** The prevalence of dippers (orange) was higher in the upper-thoracic spinal cord injury (UT-SCI) and non-injured control (NI-C) groups compared to the cervical spinal cord injury (C-SCI) group, where non-dippers (blue) and reverse dippers (green) were more prevalent. Extreme dippers (purple) were most common in the NI-C group. There was a statistically significant association between the SBP nocturnal dip categories and the C-SCI, UT-SCI, and NI-C groups ($\chi^2 = 29.422$, $P < 0.001$). **(B) *Classification of DBP Nocturnal Dippers:*** The proportion of extreme dippers was highest in the NI-C group, whereas the C-SCI group showed a higher prevalence of reverse dippers and non-dippers. The UT-SCI group had a relatively higher proportion of dippers compared to the C-SCI group. There was a statistically significant association between the DBP nocturnal dip categories and the C-SCI, UT-SCI, and NI-C groups ($\chi^2 = 30.642$, $P < 0.001$).

Supplementary Figure 3

The URP-CTREE identified homogeneous subgroups based on group (i.e., C-SCI or UT-SCI and NI-C) for all BPV indices (i.e., SD, CoV, ARV, and VIM) of SBP over the 24-hour period (Supplementary Figure 2. A-D). The importance of predictors was assessed using variable importance measures (i.e., how much the variable contributes to the predictive power of the model) for SD (5.50), CoV (0.0014), ARV (3.377), and VIM (3.695), overall indicating that NLI is strongly associated with changes in these BPV indices and the only variable that met the significance criterion for splitting. Furthermore, NLI as the most important predictor was based on the root mean square error (RMSE; how far predictions are from actual values) and coefficient of determination (r^2; how well the model explains variation in the data) for SD (RMSE = 4.64, r^2 = 0.28), CoV (RMSE = 0.038, r^2 = 0.31), ARV (RMSE = 3.137, r^2 = 0.32), and VIM (RMSE = 4.425, r^2 = 0.26), demonstrating a moderate predictive accuracy.

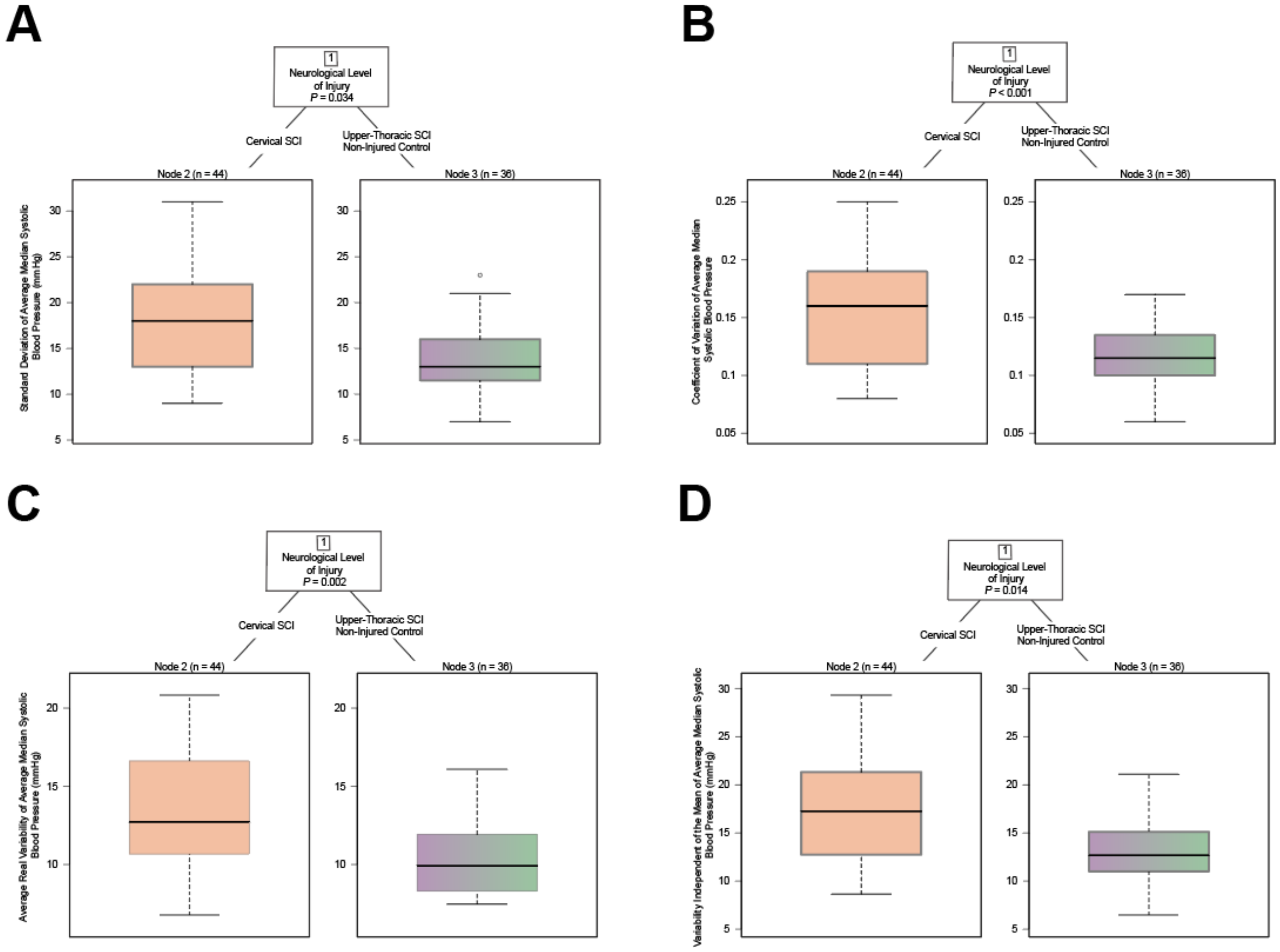

Supplementary Figure 3. Decision tree models for systolic BPV indices. Conditional inference trees, using a broad set of demographics (i.e., biological sex and age), injury characteristics (i.e., neurological level of injury, injury severity, and time since injury) and study as predictors are displayed over the 24-hour period for **(A)** standard deviation for SBP, **(B)** coefficient of variation for SBP, **(C)** average real variability for SBP, and **(D)** variability independent of the mean for SBP. The upper part represents the sequential splits based on neurological level of injury (cervical spinal cord injury versus upper-thoracic spinal cord injury and non-injured control). Boxplots at the bottom show sample size and distribution of the respective labelled systolic BPV indices within each subgroup (node).
